# Microbiological insight into various underground gas storages in Vienna Basin focusing on methanogenic *Archaea*

**DOI:** 10.3389/fmicb.2023.1293506

**Published:** 2023-12-13

**Authors:** Nikola Hanišáková, Monika Vítězová, Tomáš Vítěz, Ivan Kushkevych, Eva Kotrlová, David Novák, Jan Lochman, Roman Zavada

**Affiliations:** ^1^Section of Microbiology, Department of Experimental Biology, Faculty of Science, Masaryk University, Brno, Czechia; ^2^Department of Agricultural, Food and Environmental Engineering, Faculty of AgriSciences, Mendel University in Brno, Brno, Czechia; ^3^Department of Biochemistry, Faculty of Science, Masaryk University, Brno, Czechia; ^4^Innovation Unit, NAFTA a.s., Bratislava, Slovakia

**Keywords:** methanogens, methane, hydrogen storage, methanation, *Archaea*

## Abstract

In recent years, there has been a growing interest in extending the potential of underground gas storage (UGS) facilities to hydrogen and carbon dioxide storage. However, this transition to hydrogen storage raises concerns regarding potential microbial reactions, which could convert hydrogen into methane. It is crucial to gain a comprehensive understanding of the microbial communities within any UGS facilities designated for hydrogen storage. In this study, underground water samples and water samples from surface technologies from 7 different UGS objects located in the Vienna Basin were studied using both molecular biology methods and cultivation methods. Results from 16S rRNA sequencing revealed that the proportion of archaea in the groundwater samples ranged from 20 to 58%, with methanogens being the predominant. Some water samples collected from surface technologies contained up to 87% of methanogens. Various species of methanogens were isolated from individual wells, including *Methanobacterium* sp., *Methanocalculus* sp., *Methanolobus* sp. or *Methanosarcina* sp. We also examined water samples for the presence of sulfate-reducing bacteria known to be involved in microbially induced corrosion and identified species of the genus *Desulfovibrio* in the samples. In the second part of our study, we contextualized our data by comparing it to available sequencing data from terrestrial subsurface environments worldwide. This allowed us to discern patterns and correlations between different types of underground samples based on environmental conditions. Our findings reveal presence of methanogens in all analyzed groups of underground samples, which suggests the possibility of unintended microbial hydrogen-to-methane conversion and the associated financial losses. Nevertheless, the prevalence of methanogens in our results also highlights the potential of the UGS environment, which can be effectively leveraged as a bioreactor for the conversion of hydrogen into methane, particularly in the context of Power-to-Methane technology.

## 1 Introduction

Underground gas storage (UGS) facilities are crucial as gas infrastructure objects used to store natural gas. Natural gas is stored in the reservoir during periods when demand for natural gas is low, and then injected back into the gas grid during periods when demand for gas is high. More than 250 UGSs are in operation in Europe, and most of them are depleted oil and gas fields (GIE, 2021). These UGSs provide a storage capacity of 1572.2 TWh.

There are three types of UGSs (Molíková et al., 2022): depleted hydrocarbon reservoirs, aquifers and salt caverns. Depleted hydrocarbon reservoirs are the most suitable for underground gas storage because they have well-defined parameters and geological structure, with an impermeable layer of caprock on top and they are surrounded by underground water. Aquifers resemble depleted hydrocarbon storages in that they have a porous rock structure filled with saline or fresh water. Aquifers for storage purposes should form an anticlinally deposited space in the underground, the top of which must be rock of sufficient strength to prevent leakage of the stored gas. The third type, salt caverns, are artificially created structures specifically designed for gas storage. They are made using the technology known as solution mining, which involves pumping water into the salt structure and collecting the brine obtained by dissolving the salts in the cavern. In this case, the shape of the reservoir and the surrounding rock must also be studied to evaluate whether the reservoir can be used for gas storage (Falzolgher and Altieri, 2005).

Gas has been stored for decades. Before natural gas was used, town gas was stored and injected into underground storage (Šmigáň et al., 1990; Buzek et al., 1994). Town gas is a gas mixture produced during the gasification of coal, also known as coal gas, with a varying composition of about 50% H_2_, 30% CH_4_, 10% CO_2_, 2% N_2_ and 10% CO (Buzek et al., 1994; Hiller et al., 2006). Storage of town gas in natural storage of aquifer character in Lobodice resulted into changes in the volume fraction of stored gases, as well as in their volume. The volume fraction of hydrogen decreased, while the volume fraction of the methane increased. Based on calculations and isotopic analyses, as well as cultivation experiments, methanogenic archaea responsible for the conversion of hydrogen to methane were found in the underground water samples (Šmigáň et al., 1990; Buzek et al., 1994). This work follows the research of Šmigáň et al. (1990) and Buriánková et al. (2022), who studied microbial composition in UGS. Hydrogen conversion is not the only phenomenon that can occur in UGS. Carbon dioxide storage has also led to an increase in the microbial population and its activity, shifting from a chemoorganotrophic to a chemolithotrophic community (Morozova et al., 2010, 2011; Gniese et al., 2014).

The presence of live, active microorganisms in UGS is an important factor that can be perceived positively or negatively depending on the main objective of using UGS. One of the possible applications is Power-to-Gas technology (Wulf et al., 2020). This technology is based on the conversion of excess electrical energy into gas that can be stored and used later, e.g., hydrogen or methane. Methanogenic archaea, which have been repeatedly confirmed in underground environments (Godsy, 1980; Kotelnikova et al., 1998; Ivanova et al., 2007; Buriánková et al., 2022), are excellent candidates for this technology because they are able to convert molecular hydrogen and carbon dioxide into methane. This could lead to a reduction of CO_2_ emissions and generate new green energy through microorganisms. The verification of the presence of methanogens within underground gas storage (UGS) facilities, coupled with the provision of favorable environmental conditions, represents a pivotal prerequisite for the effective deployment of the Power-to-Methane technology. This technology relies on delivering green hydrogen and carbon dioxide (CO_2_) to methanogens present in UGS in the correct ratio. This can lead to successful implementation of methanogenesis *in situ*, as it was demonstrated by Vítězová et al. (2023), and production of green CH_4_.

Simultaneously, hydrogen storage and planned gradual increase of hydrogen concentration in natural gas are now the focus of the energy sector, as they correspond to current trends and future prospects (Aftab et al., 2022; Cristello et al., 2023; Galyas et al., 2023). The development of a hydrogen economy is one of the key pillars of the European Green Deal. Hydrogen is a green, versatile energy carrier that produces no greenhouse gases when burned. It can also be used to store excess renewable energy, such as solar and wind power, through Power-to-Gas technology (Wulf et al., 2020). This stored energy could be later used to generate electricity or heat. Due to fluctuating electricity demand, the hydrogen needs to be stored during periods of low demand and withdrawn during periods of high demand, as is the case with natural gas. However, enormous amounts of stored hydrogen are needed to meet energy demand and supply electricity for whole populations. UGSs provide such an environment with high storage capacity (GIE, 2021). However, hydrogen as a gas has different properties compared to natural gas or methane, for example, lower density, molecular size and good permeability through various materials (Meda et al., 2023).

For the purpose of hydrogen storage in the UGS, the presence of microorganisms and conditions in UGS must be considered, as there are a number of autotrophic microorganisms that can consume hydrogen. This could result in substantial losses of stored hydrogen, thereby incurring associated economic losses (Dopffel et al., 2021; Perera, 2023). Not only methanogens that utilize hydrogen are present in the UGS, but also other microorganisms that use this molecule, such as acetogenic and sulfate-reducing bacteria (SRB). The conditions in the UGS would be advantageous for these groups of microorganisms because of the high partial pressure of the substrate gas in the UGS. The carbon required for the reaction could be acquired from dissolved carbonates from the bedrock, which could enable release of deposited CO_2_ into the UGS environment (Bo et al., 2021; Dopffel et al., 2021). Potential hydrogen leaks from underground reservoirs are often discussed in connection with hydrogen storage (Aftab et al., 2022). Possible hydrogen losses during storage in UGS are not yet well described. Conditions in UGS are difficult to simulate due to many variable parameters, although many researchers are focusing on characterizing and modeling UGS environments (Heinemann et al., 2021; Thaysen et al., 2021; Aftab et al., 2022; Hogeweg et al., 2022; Dohrmann and Krüger, 2023; Strobel et al., 2023; Tremosa et al., 2023). From known information about isolated methanogenic species, it appears that the methanogenesis would not occur under extreme UGS conditions, such as high salinity (2.5 M and above), high temperature (above 122°C) and extreme pH (pH < 4.5 or pH > 10) (Thaysen et al., 2021).

The three groups of microorganisms mentioned above are also studied for their direct or indirect effects on corrosion (Li et al., 2018; Dopffel et al., 2021). SRB have been repeatedly confirmed to cause corrosion (Dinh et al., 2004; Enning et al., 2012); acetogenic bacteria can also cause microbial corrosion (Kato et al., 2015; Philips et al., 2019), and methanogens are highly expected to cause corrosion with two species having been confirmed for this ability (Dinh et al., 2004; Uchiyama et al., 2010; Hirano et al., 2022). The mechanism of Microbiologically Influenced Corrosion (MIC) is not yet fully understood, as different microorganisms are involved and it could also depend on pure cultures or consortia (Liang et al., 2019; Procópio, 2022). It is not yet known how they behave in the presence of hydrogen and how the corrosion rate could be reduced.

In this study, we present microbial communities in UGS, their composition in different UGSs in Vienna Basin and confirmation of their viability by cultivation. We focused on methanogenic archaea and SRB, as these are the most important groups in terms of potential methanation, hydrogen storage and also corrosion threat. We provide complex analysis of 16S rRNA sequencing and support the obtained data with results from cultivation to prove viable microorganisms in the UGS environment.

Detecting methanogenic DNA in a sample is crucial, but it’s equally important to ascertain the presence of viable microorganisms. Relying solely on DNA sequencing results does not allow us to confirm the existence of living microorganisms. Cultivation methods are essential for not only identifying the species within the samples but also for uncovering methanogens that might exist in low quantities in their original environment. These low-abundance methanogens might be overlooked due to biases in sample processing or data analysis. Moreover, if living methanogens are present in limited numbers in the initial sample, they can potentially proliferate when provided with the appropriate substrate. For hydrogenotrophic methanogens, this substrate is hydrogen, as suggested by Vítězová et al. (2023). That phenomenon has important implications for applications such as hydrogen storage and methanation, and highlights the need for the use of cultivation methods.

Because knowledge of UGS microbial composition and similarities is important to model and predict gas storages behavior, we have collected 16S rRNA sequencing data from various underground environments worldwide to perform complex analysis of microbial communities.

## 2 Materials and methods

### 2.1 Sampling site

Water from UGS was sampled from 7 objects throughout the Vienna Basin in the Czech Republic and Slovakia. All UGSs belong to the type of depleted gas reservoirs. These seven objects could be divided across locations. The first six objects are located in Slovakia, in the east of Vienna Basin, near the borders with Austria. Objects 1, 2 and 3 are located nearby, as are objects 4, 5 and 6. The last UGS, object 7, is located in the north of the Vienna Basin in the Czech Republic. Conditions such as temperature, depth and physicochemical parameters differed from each other (Table 1).

**Table 1:** Representative parameters for all sampled objects.

	Object 1	Object 2	Object 3	Object 4^*^	Object 5^**^	Object 6	Object 7
Depth [m]	656	1952	753	1,060	640	653.3	1738
Temperature [°C]	28	70	44	44	35	33	56
Salt content [mg/l]	10987.4	21622.0	19088.8	14217.5	25036.9	15360.9	11378.0
Sulfates [mg/l]	1.9	3.0	2.0	20.6	57.2	2.0	3.0
Hydrogen carbonates [mg/l]	110.9	12.2	1005.0	614.9	1271.2	945.8	335.6
Carbonates [mg/l]	178.0	168.0	–	–	–	90.0	168.0
pH [−]	8.95	9.11	7.62	7.5	7.7	8.46	9.02

*data measured in 2011.

**data measured in 1992.

### 2.2 Media and culture conditions

The experiment was focused on two groups of organisms and confirmation of their presence as living organisms in the environment. Different media were prepared for their cultivation. To confirm the presence of viable SRB in the sample, Postgate C medium (Postgate, 1984) supplemented with Mohr salt to final concentration of 1% was used for their cultivation and the pH was adjusted to 7 (Supplementary Table S1). Three types of media were used to support growth of the different methanogenic microorganisms. The modified freshwater and saltwater medium (Widdel and Bak, 1992) and the DSMZ 141 Methanogenium medium. The first medium was used to support the growth of methanogens, which grow better in a low-salinity environment; the other two are more suitable because the salinity is consistent with that in the UGS. The DSMZ 141 Methanogenium medium is rich in nutrients compared to the saltwater medium and could support growth of more methanogens, but also a rich spectrum of bacteria. See Supplementary Table S2 for the full media recipes.

### 2.3 Sampling of water

The water samples collected from the UGS were of two types; deep water samples (tagged by letter D) and water collected either from gas separators or riser pipes (tagged by letters S and R). Gas separators are a component of surface technology used to eliminate water and potential crude oil contaminants from stored natural gas. A riser pipe is a vertical conduit or pipe used to transport natural gas from its source, such as an UGS or offshore well, to the surface or to another part of the gas distribution system or to transfer injection fluids, control fluids or lift gas from the surface facilities to UGS. Only sample D was taken from object 3. Deepwater samples were obtained using a sterile sampler, which was lowered via riser pipes to the specific horizon within the underground gas reservoir, and water was extracted from that horizon (depth). Once the sampler reached the surface, the water was depressurized and immediately transferred into 1 L sterile sample containers, without the gas phase. The water was kept overnight at 4°C before being taken to the laboratory for processing. The water from separators was filled into sterile containers with a volume of 15 L and also stored at 4°C before being taken to the laboratory. After delivery, the physical parameters of the water samples were measured (Supplementary File 2).

### 2.4 Cultivation of microorganisms

#### 2.4.1 Cultivation of methanogens

The prepared media were immediately inoculated with sampled water under aseptic conditions in Laminar Box, Herasafe KS (Thermo Fisher Scientific, MA, USA). Cultivation was performed in sealed serum bottles of 120 mL volume filled with 30 mL of medium. 3 mL of the water sample was inoculated into a serum bottle containing 30 mL of media for methanogens. The gas phase was purged with the gas mixture H_2_:CO_2_ (4:1, v/v) (gas mixer, WITT-Gasetechnik GmbH & Co KG, Witten, Germany); (H_2_ pur. ≥99.8%, CO_2_ pur. ≥ 99.5%, SIAD Czech spol. s r.o., Rajhrad, Czech Republic) and then pressurized to 0.3 MPa absolute pressure. Three different media were used, each inoculated in triplicate.

#### 2.4.2 Cultivation of sulfate-reducing bacteria

The medium for SRB was dispersed into 2 mL microtubes and inoculated with 200 μL of the water sample. The overfill of media in the tubes ensured that no gas phase was present. Samples were cultivated at a temperature equal to that measured in the UGS (Supplementary File 2, the temperature measured at the place of the perforation in the well of the UGS). Water samples collected from surface technologies were cultivated at lower temperature, or two temperatures for cultivation were set to support growth of present microorganisms (Supplementary File 2).

The water samples were also observed microscopically for the presence of autofluorescence immediately after delivery to the laboratory, and DAPI staining was also performed.

### 2.5 Isolation of pure cultures

#### 2.5.1 Methanogens

For samples where growth was detected by a pressure drop in the serum bottles and also microscopically (autofluorescence), cultures were reinoculated into media containing an antibiotic mixture of vancomycin, ampicillin and kanamycin at a final concentration of 100 μg/mL each to suppress the bacteria present.

To isolate pure cultures, the agar dilution tubes technique was used (Hanišáková et al., 2022). For this purpose, 3.3 g of agar BD Difco™ (Becton, Dickinson and Company, Franklin Lakes, NJ, USA) was washed five times to remove impurities, then dissolved in 100 mL of deionized water and dispersed à 3 mL into test tubes, which were previously autoclaved at 121°C; 20 min. The liquid agar was placed into water bath BM 15 (NÜVE, Ankara, Turkey) and tempered to 60°C. 6 mL of the medium was pipetted into test tubes and tempered to 45°C. The 6 test tubes were placed into anaerobic box (Coy Laboratory Products, Grass Lake, MI, USA) and dilution series were prepared, with 1 mL pipetted into the first test tube. The tubes were left in the anaerobic box overnight to adjust the anaerobic conditions in the media. The tubes were sealed with a butyl stopper and taken out of the anaerobic box to replace the gas phase with H_2_:CO_2_ mixture (4:1, v/v). The test tubes were incubated in upside down position at the appropriate temperature. After 14–30 days of cultivation, the visible colonies were picked out with long needles (B.Braun Sterican Needles 21G x 4.75, Ø0.80 × 120 mm) and suspended in 1 mL of medium in the microtube, which was immediately inoculated into fresh medium in the serum bottle. Serum bottles that showed microbial growth were checked for purity under the microscope and DNA was isolated for culture identification.

#### 2.5.2 Sulfate-reducing bacteria

Sulfate-reducing bacteria produce hydrogen sulfide. The presence of hydrogen sulfide in the medium is detected by the formation of a black sodium sulfide precipitate due to the presence of Mohr’s salt. Microtubes that were positive for hydrogen sulfide production were further purified to obtain SRB pure cultures. The positive samples were serially diluted in microtubes. 100 μL of the diluted sample was mixed with 25 mL of tempered Postgate C medium supplemented with agar (1.2%) and poured anoxically into sterile Petri dishes. Petri dishes were placed into the anaerobic jar Anaerostat DIAB 10001 (Dinkelberg analytics, Gablingen, Germany) with anaerobic atmosphere generator Thermo Scientific™ Oxoid™ CO_2_ Gen™ Sachet (Thermo Fisher Scientific, Waltham, MA, USA) and cultivated at the desired temperature. Black-stained colonies that formed in the agar were picked out with a needle and inoculated into fresh medium. Positive samples were checked for purity and further analyzed.

### 2.6 Microscopy

The presence of methanogens in the sample and verification of their apparent purity were determined using an Olympus BX50 fluorescence microscope equipped with the Olympus U-RFL-T power supply (Olympus Life Sciences, Japan) and the U-MBW2 fluorescence filter cube for autofluorescence and U-MWU for DAPI staining (Olympus Life Sciences, Japan). Cells were observed under phase contrast for growth control and autofluorescence. Autofluorescence and DAPI staining were performed for initial observation of the samples. 6 mL of the deep water sample and 10 mL of the surface technology water sample were filtered through the 0.45 μm membrane Isopore^tm^ (Merck, Darmstadt, Germany). Cells captured on the membrane were stained with DAPI (60 μg/mL) and then incubated at 4°C for 15 min. After incubation, the membrane was washed in three baths as follows: deionized water, ethanol and deionized water again. The membrane was observed under a microscope.

### 2.7 DNA isolation and PCR amplification

DNA of pure microbial cultures, obtained by cultivation techniques for identification, was isolated by DNeasy UltraClean Microbial Kit (Qiagen GmbH, Hilden, Germany). The 16S rDNA gene was amplified by PCR technique using the specific primers for methanogens 344af 5’-ACGGGGYGCAGCAGGCGCGA-3′ (Casamayor et al., 2002) and 1406uR 5’-ACGGGCGGTGTGTRCA-3′ (Eder et al., 1999) resulting in a PCR product of length 1000 kb. For sulfate-reducing bacteria, universal primers for bacteria were used, 8FPL 5′-AGTTTGATCCTGGCTCAG-3′ and 806R5’-GGACT ACHVGGGTWTCTAAT-3’ (Apprill et al., 2015) giving product length of 800 kb. The PCR reaction in both cases contained 12.5 μL of PPP mastermix (Top-Bio s.r.o., Vestec, Czech Republic), 9.5 μL of ultrapure water, 1 μL (10 μM) of each primer and 1 μL of the DNA sample. The program for primer pair 344af/1406uR began with an initial denaturation at 96°C for 1.5 min, followed by 10 cycles of 96°C for 30 s, 60°C for 30 s and 72°C for 60 s, and then 25 cycles of 94°C for 20 s, 60°C for 30 s and 72°C for 60 s, terminating with a final step at 72°C for 10 min. The program for primer pair 8FPL/806R began with an initial denaturation at 94°C for 5 min, followed by 35 cycles of 94°C for 30 s, 53°C for 30 s and 72°C for 60 s, terminating with a final step of 72°C for 10 min. Successful amplification of DNA was checked by performing electrophoresis and the purified PCR product MinElute PCR Purification Kit (Qiagen GmbH, Hilden, Germany) was sent to a commercial laboratory for sanger sequencing (Eurofins genomics, GmbH, Vienna, Austria). The sequences were compared with the GenBank database using the algorithm BLAST, omitting sequences from environmental samples, to identify the isolates by comparison to already cultivated and identified strains.

### 2.8 Next-geneartion sequencing

#### 2.8.1 Sample preparation

To obtain DNA for whole microbial community analysis, the water sample was prefiltered using cellulose filter 80 g/m^2^ (P-LAB, Praha, Czech Republic), to reduce the number of inorganic particles that could slow down the next filtration step. The prefiltered water was further filtered through a 0.2 μm membrane filter Isopore^tm^ (Merck, Darmstadt, Germany). The volume of filtered water was recorded. DNA from the filter was isolated using the DNeasy Power Water isolation kit (Qiagen GmbH, Hilden, Germany) and further processed using the Illumina MiniSeq sequencer.

The V4 hypervariable region of the 16S rRNA gene sequence was targeted by single-step PCR using the modified 515F and 806R primer pair (Pichler et al., 2018). PCR amplification was carried out using a 0.8× concentration of Platinum II Taq Hot-Start DNA polymerase, a final primer concentration of 200 mM, and 2 μL of template DNA in a total volume of 25 μL. The amplification profile consisted of an initial step at 95°C for 3 min, followed by 35 cycles of denaturation at 94°C for 20 s, annealing at 55°C for 30 s (50% thermal ramp), extension at 72°C for 30 s, and a final extension step at 72°C for 5 min. After PCR, amplified products were purified with Agencourt® AMPure XP beads at 0.8× concentration. Samples were then manually normalized and pooled using the Qubit 4.0 fluorometer (Thermo Fisher Scientific, Waltham, MA, USA) and QuantiFluor® dsDNA System (Promega, Madison, WI, USA). Verification of the normalized PCR amplicons was carried out after pooling using Roche LightCycler 480-II (Roche, Switzerland) with KAPA Library Quanitification Kit and Qubit 4.0 fluorometer together with QuantiFluor® dsDNA System. The amplicon size distributions of the pools and the final library were checked using Fragment Analyzer (Agilent, Santa Clara, CA, USA) with HS NGS Fragment Kit (DNF-474). The library was sequenced using MiniSeq (Illumina, San Diego, CA, USA) with MiniSeq Mid Output Kit (300 cycles).

#### 2.8.2 Data analysis

The raw fastq files were processed using DADA2 in R according to the standard operating procedure. Briefly, the reads were first filtered and trimmed. Filtered reads were then de-replicated and de-noised. Reads were then merged, chimeras removed, and taxonomic assignment performed using the RDP naive Bayesian classifier method against Silva v138.1 database.

Results of 16S rRNA composition of microorganisms in the samples were further analyzed by R statistics (v4.2.3.) using Rstudio (v2023.03.0) microeco R package (v0.20.0) (Liu et al., 2021). Alpha diversity (Chao1, ACE, Shannon, Simpson) and beta diversity (Bray-Curtis and Jaccard) were calculated and analyzed. Prediction of the functional profile of communities present in the samples was analyzed using the FAPROTAX database (Louca et al., 2016). FAPROTAX database allows the prediction and generation of functional profiles and ecological functions based on the assignment of metabolic functions to specific taxon. This prediction was used in several studies (Sansupa et al., 2021; Yang et al., 2022) and helps to better understand microbial community structure. Although, the analyzed data were extracted from DNA and not RNA, which would better reflect the current functional activity in the community. In addition to FAPROTAX, which is based on the prediction of metabolic pathways based on the taxa assignment performed, data were also analyzed using functional prediction using PICRUSt2 (Phylogenetic Investigation of Communities by Reconstruction of Unobserved States) (v2.5.2) (Douglas et al., 2020). PICRUSt2 output data were analyzed through microeco (Liu et al., 2021) and the ggpicrust2 R package (v1.7.1) (Yang et al., 2023).

### 2.9 Global terrestrial subsurface microbiome analysis

The analysis aimed at comparing different microbiomes from the terrestrial subsurface proceeded according to the following steps. Publications dealing with the microbial composition of the subsurface were searched using various scientific platforms and their search algorithm, cross-references of the publications dealing with UGS issues, and keywords, such as Underground gas storage, hydrogen storage, UGS microbiome and more. Since we have been working on UGS and hydrogen storage for a long time, we collected a number of publications that were also used as data source. The publications were reviewed and sorted, and additional information on the samples was extracted into metadata (Supplementary File 3). The criteria for inclusion of data into analysis were: analysis of the microbiome through 16S rRNA sequencing and publicly available raw data. If more wells were sampled in the publication, only a few representatives were selected. Raw sequences were retrieved through SRA numbers associated with responding BioSample. Since the samples differed depending on the primer set and layout strategy used, they had to be processed separately. The processing was performed in R through Rstudio, using the DADA2 package and associated pipeline. The filtered sequence tables obtained after the process were merged after removal of chimeras and assignment to taxonomy. The sequences were assigned according to the latest release of Silva database (v138.1). If the domains were sequenced separately, they were excluded from the overall comparison, but included in the analysis of the specific domain so that three data sets were analyzed separately for comparison. The analyses were processed using R package phyloseq (McMurdie and Holmes, 2013) and microeco (Liu et al., 2021). The tax_glom function was used to merge taxa at the genus level. From the processed data, alfa and beta diversity were calculated and non-metric multi-dimensional scaling (NMDS) was calculated and plotted using the Bray-Curtis distance method. Spearman’s correlation coefficients between environmental factors or underground sample type and taxa abundance or predicted pathway abundance were calculated, with the value of *p* adjusted using the FDR method; and heatmaps were constructed from the data.

## 3 Results

### 3.1 Microscopic observation

Samples were observed under the microscope as native preparations or DAPI stained fluorescent preparation. The autofluorescence of the samples was confirmed only in samples S1, S5 and D3 (Supplementary Figure S1). Negative autofluorescence in the other samples could be due to low microbial density, so the probability of a methanogenic cell is low. Morphological profile of the specimens stained with DAPI differed depending on the object and variant (Supplementary Figure S2). Deep water samples generally had a lower abundance of cells, which corresponded with the duration of filtering the water samples for molecular biological analysis. The morphology of cells included small rods, longer rods or thick rods in chains, also cocci of various sizes and cell bundles of *Methanosarcina*-like aggregates in samples S1, S4 and S5. In samples S2 and R7, the morphology is monotonous compared to the other samples. Sample D5 was not successfully stained and is therefore not included in the overall view of DAPI stained samples in Supplementary Figure S2.

### 3.2 Microbial communities

Microbial profiles of the individual samples differed more between the deep and surface technology samples than between the individual sampled objects (Supplementary Figures S3, S4). The sample from riser pipes was the most distant from the other samples, while the deep water samples from the depleted field clustered together, except for sample D3 (Supplementary Figure S4). The uniqueness of the samples and taxa is also evident form the Venn diagram (Supplementary Figure S5). The prevalence of archaeal 16S rRNA copies was quite high, accounting for more than 50% of the total 16S rRNA in five samples (Supplementary Figure S6). The highest number of archaeal 16S rRNA copies was found in sample S1 (87%). In contrast, the lowest number of archaeal 16S rRNA was found in sample R7 (4%), which was collected from riser pipes. This sample was the most different from all other samples, and also had the lowest value in observed, ACE and Shannon alpha diversity index (Supplementary Table S3; Supplementary Figure S3). Low diversity was also observed in samples S1, S4 and S5. On the other hand, the most diverse samples were the deep water samples from reservoir, with sample D5 having the highest values of all alpha diversity indexes (Supplementary Table S3).

#### 3.2.1 Archaea

**Figure 1:**
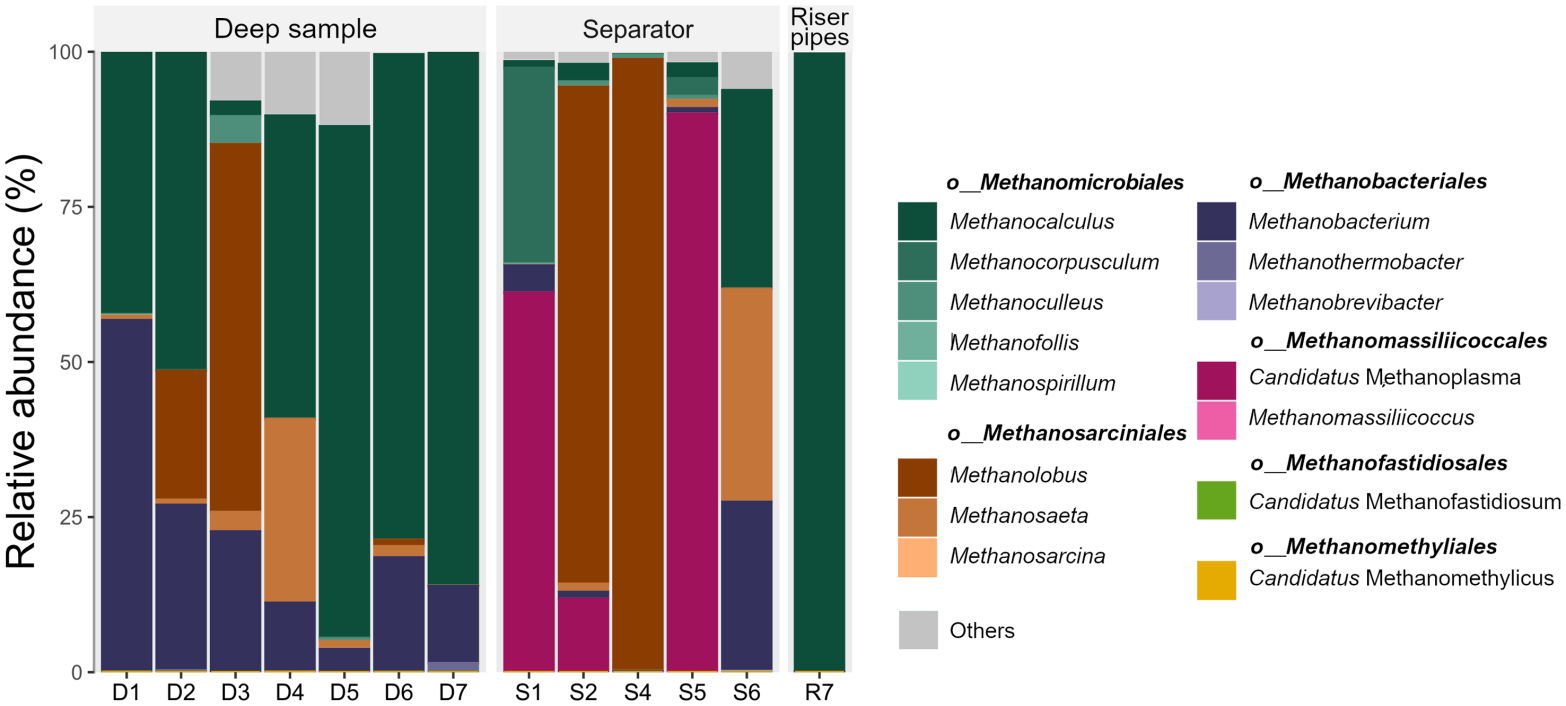
Relative abundance of the most abundant archaeal genera and their affiliation to archaeal orders in each sample. D, Deep sample; R, Riser pipes; S, gas separator.

Operation of UGS technologies very often requires the injection of methanol into the well. Methanol plays two important roles: it prevents the water in the well from freezing and it prevents the formation of gas hydrates. Methanol is a compound that serves as one of the most important substrates in methanogenesis, namely in the methylotrophic metabolic pathway. The presence of methanol leads to the formation of strong methylotrophic methanogenic communities in surface technology. High prevalence of the genus *Methanolobus* and “*Candidatus* Methanoplasma” in surface samples indicates that the “feeding” of microbial consortia in UGS with methanol can lead to overgrowth of this group of microorganisms (Figure 1). This is evident in sample S1, where “*Candidatus* Methanoplasma” presents 53% of all sequenced 16S rRNA, in sample S5 it is even 59%; and in sample S4 the genus *Methanolobus* consists of 64% of all microbial 16S rRNA. The presence of DNA from methanotrophic bacteria was not confirmed.

The methylotrophic pathway, which is either hydrogen-dependent or independent, is dominant in methanogenic pathways in surface technologies. In comparison, in the deep water samples in the UGS, methanol supply is insignificant as a carbon and energy source and the dominant pathway is mostly hydrogenotrophic methanogenesis. *Methanomicrobiales* is the dominant order in almost all deep samples, followed by the order *Methanobacteriales* (Figure 1). Order *Methanomicrobiales* includes species that grow in variable conditions, mesophilic and thermophilic environments and also in marine environments. They are also commonly isolated from oil fields and anaerobic digesters (Ollivier et al., 1998; Cheng et al., 2008; Weng et al., 2015). Representatives of the order *Methanomicrobiales* are able to grow in environments with higher salt concentrations. Therefore, their presence in deep water samples is not so surprising. *Methanocalculus* was characteristic and dominant in most deep water samples, while the representatives of the order *Methanomicrobiales* present in surface technology samples (gas separators and riser pipes) were *Methanoculleus*, *Methanocorpusculum* and *Methanofollis* (Figure 1; Supplementary Figure S7).

Sample D3 differs from the other samples in the high abundance of the genus *Methanolobus* and the higher abundance of the genus *Methanobacterium*. The dominance of methylotrophic methanogens instead of hydrogenotrophic ones could indicate the leakage of hydrocarbon compounds into the deep/reservoir water. The presence of *Methanosaeta* sp. in the deep water samples and in sample S6 is also interesting. This could indicate the importance of acetoclastic pathway over methylotrophic in subsurface environment (Figure 1; Supplementary Figure S7). Rare methanogenic taxa present at low abundance, as “*Candidatus* Methanofastidiosum” and “*Candidatus* Methanomethylicus” were detected only in deep water samples, with an abundance of archaeal 16S rRNA <0.1%.

#### 3.2.2 Bacteria

**Figure 2:**
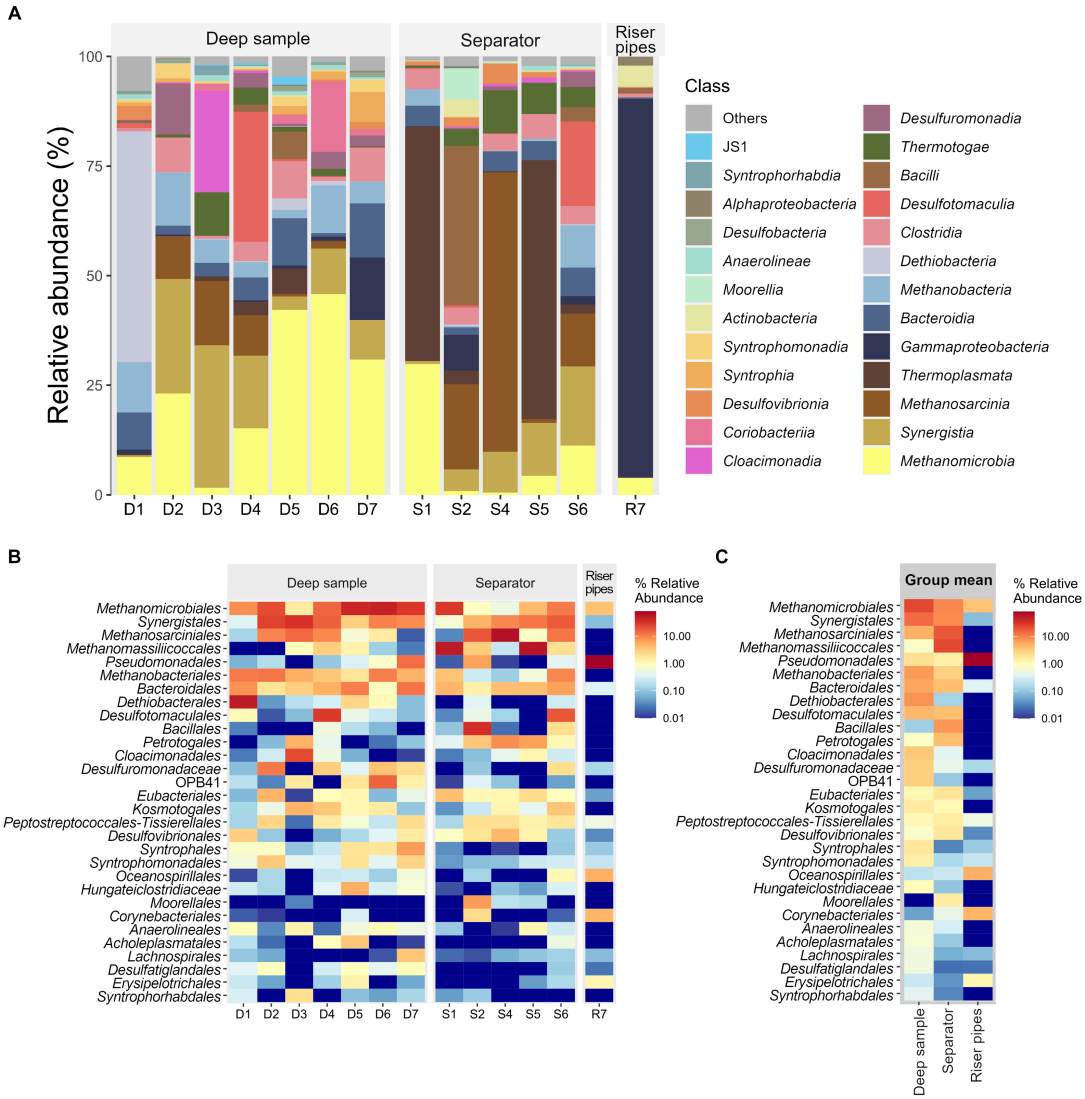
**(A)** Relative abundance of the most abundant microbial classes in each sample. **(B)** Heatmap of the thirty most abundant microbial orders in the samples. Individual samples separated by sample type. **(C)** Group mean abundances subdivided by sample type.

The occurrence of hydrogenotrophic methanogens in the UGS is only possible if hydrogen is present in the environment. Without hydrogen, only methylotrophic and acetoclastic pathways would dominate. Fermentation is an important and necessary step to meet the hydrogen demand in the UGS. The presence of hydrogen-producing fermentative microbial groups generates sufficient hydrogen for methanogens. Petroleum hydrocarbons, which are often present in UGSs, are anaerobically oxidized and fermented by syntrophic microorganisms that depend on the methanogens as they keep hydrogen level low. Figure 2B shows a high prevalence of various syntrophic, hydrogen-producing microorganisms such as *Syngergistales*, *Syntrophomonodales*, *Syntrophales* and other fermenting hydrogen-producing groups such as *Bacteroidales* and *Eubacteriales*. Fermenting, hydrogen-producing microorganisms of phylum *Thermotogota* were also present in the samples in varying amounts of up to 10% of 16S rRNA copies (Figure 2A).

The abundance of sulfate-reducing bacteria potentially associated with microbially influenced corrosion of iron and steel varied among samples and consisted of different populations of these microorganisms. The genus *Desulfovibrio* (order *Desulfovibrionales*) was the most abundant SRB in the surface technologies samples and accounted for 3 to 13% of the bacterial 16S rRNA, with the exception of samples S6 and R7 (Figures 2B,C). A relatively high abundance of SRB was detected in sample S6, but with the dominance of the order *Desulfotomaculales*, which accounted for 19% of the total 16S rRNA (Figure 2B). R7, on the other hand, contained no SRBs at all.

Interestingly, the deep water samples were not as consistent in the presence of SRBs. In sample D1, 3% of all microbial 16S rRNA copies were representatives of the order *Desulfovibrionales,* and 50% of all 16S rRNA copies belonged to *Dethiobacter* sp. (class *Dethiobacteria*) (Figure 2A). This genus, with only one known culturable species, *Dethiobacter alkaliphilus*, is not sulfate-reducing but can reduce thiosulphate, elemental sulfur and polysulphide with H_2_ (Sorokin and Merkel, 2022). The *Dethiobacteria* class was also detected in sample D5, although its abundance was only 3% of all 16S rRNA copies. A large amount of SRB was detected in sample D4, with 30% of all 16S RNA copies belonging to the order *Desulfotomaculales* (class *Desulfotomaculia*). Members of the class *Desulfuromonadia* were also detected in the samples. They are not sulfate-reducers but they are involved in the sulfur cycle and also associated with corrosion. About 4% of the total 16S rRNA in sample D6 belonged to the class *Desulfuromonadia*, and at similar abundance, 2.5% of the total 16S rRNA in sample D7 was assigned to the class *Desulfuromonadia*, followed by 1% of abundance of *Desulfovibrionia* and *Desulfobacteria*. Higher abundance, 11% of the total 16S rRNA in sample D2, belonged to the class *Desulfuromonadia*. Less than 0.1% of all 16S rRNA copies belonged to SRB in deep sample D3.

**Figure 3:**
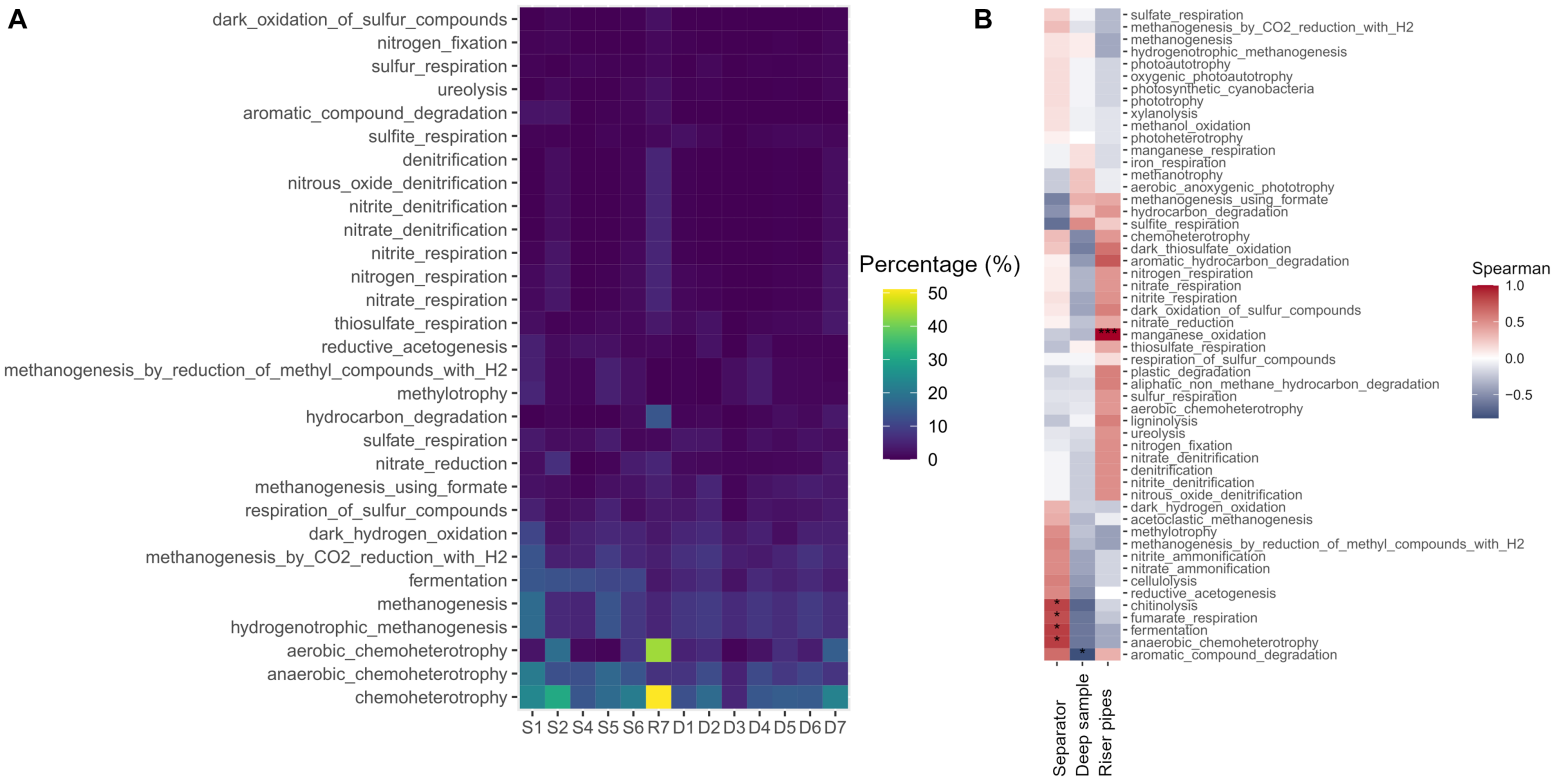
**(A)** The prediction of the metabolic functional profile FAPROTAX. **(B)** The Spearman correlation between the predicted metabolic pathway and the origin of the sample (*p < 0.05, **p < 0.01, ***p < 0.001).

In sample R7, which was the most different from all other samples 80% of the 16S rRNA belonged to the genus *Pseudomonas*, which was very abundant in this sample and was also easily seen under the microscope. The high abundance in this sample can also be seen in Figures 2, 3A,B. Bacteria belonging to genus *Pseudomonas* are commonly used for the biodegradation of aliphatic and aromatic hydrocarbons (Lalucat et al., 2006; Xu et al., 2018). UGS are often depleted oil fields that may contain residues of hydrocarbons from crude oil and also residues of fluids used in mining technologies. In an aerobic environment, these residues can be degraded by pseudomonads. This explains the high abundance of these bacterial species in the R7 sample.

### 3.3 Prediction of functional pathways

#### 3.3.1 FAPROTAX

Prediction of the functional microbiological profile of the samples using the FAPROTAX database supports the results that methanogenesis, fermentation and sulfur respiration are the three most abundant metabolic pathways in the UGS samples (Figure 3A). In the separator, the role of fermentation is even higher, and statistically significant (*p* < 0.05) (Figure 3B), based on the predicted metabolic pathways. Riser pipes with dominant *Pseudomonas* sp. in the functional profile show dominant chemoheterotrophy, along with denitrification and its partial reactions, and hydrocarbon degradation, metabolic pathways characteristic for *Pseudomonas* species. There is a significant correlation (Spearman, *p* < 0.001) between riser pipes and the manganese oxidation pathway, as this pathway can be performed by the pseudomonads, which were dominant in the sample (Figure 3B). However, the results are based on predictions and it depends on the prevailing environmental conditions and redox potential, which pathway would be performed in given conditions. There is a slight, non-significant (Spearman, *p* > 0.05) correlation between methylotrophic methanogenesis and separator samples, which corresponds to the high abundance of methylotrophic methanogens in the samples. On the other hand, the methanogenesis using formate showed a non-significant (Spearman, *p* > 0.05) correlation to the deep water samples and riser pipes, which is due to the archaeal dominance of the genus *Methanocalculus*, a formate-utilizing methanogen.

#### 3.3.2 PICRUSt2

Further metabolic pathway analysis was performed using PICRUSt2 (Supplementary Figure S8). While FAPROTAX predicts more functional metabolic pathways and their role in the environment of the system, PICRUSt2 goes deeper into the predicted individual metabolic pathways and enzymatic reactions. Supplementary Figure S8A shows the PCoA plot generated using the ggpicrust2 package and the predicted MetaCyc data showing the clustering of the different sample types. With the exception of one separator sample, the samples form distinct clusters, with the samples from riser pipes clearly different from the other samples.

The ALDEx2 statistical method with value of *p* adjustment was used to calculate the differential frequency of functional pathways (Supplementary Figure S8B) and indicate statistically significant pathways (*p* < 0.05). The largest difference is in the different ubiquinol synthesis pathways, which is due to the prevalence of aerobic *Pseudomonas* sp. in sample R7. Moreover, the genus *Pseudomonas* is known for its lipid degradation properties, which is also shown as a significant pathway in Supplementary Figure S8B. The significance of the hexitol degradation pathway in sample S2 may be caused by unusually high abundance of *Bacillus* sp., which accounts for 35% of the total 16S rRNA sequenced. *Bacillus* sp. is capable of growing on mannitol or glucitol, hence the importance of this particular metabolic pathway (Gay et al., 1983; Watanabe et al., 2003). Although the predicted pathway of mannan degradation is significant in samples S1, S4 and S5, this could be caused only by the presence of *Bacteroides* sp. and other bacteria with this functional pathway and not by the character of the investigated samples.

On the other hand, interestingly, the pathways that are statistically significant (*p* < 0.05) for the deep reservoir samples are the typical archaeal pathways important for methanogenesis. These are for example archaeal flavin biosynthesis, coenzyme M biosynthesis, which is responsible for the final step of methanogenesis, or archaetidylserine and archaetidylethanolamine biosynthesis pathways responsible for lipid membrane biosynthesis in archaea. Although the results are based on functional predictions, the significance of these pathways demonstrates their importance in such environments.

### 3.4 Cultivation results

#### 3.4.1 Methanogens

Serum bottles inoculated with the UGS water samples showed microbial growth after a few days, but autofluorescence was observed after 1 or 2 weeks of cultivation, as well as a drop in pressure in the serum bottles. Samples positive for methanogenic archaea were repeatedly reinoculated into fresh sterile medium and at least once into media containing an antibiotic mixture. Agar dilution series were prepared and after 3 to 4 weeks colonies were picked and inoculated into the fresh medium. Growing cultures of individual colonies that appeared microscopically pure were subjected to DNA isolation for further identification, otherwise further purification was required. The identified cultures are listed in Table 2.

**Table 2:** Identified methanogenic species isolated from the samples of various objects.

Object	Sample	Number of isolates	Closest culturable representative	BLAST identity percentage (%)	Accesion numbers	Reference accession number
1	D1	5	*Methanocalculus pumilus* MHT-1	98.97–99.59	OR227305-OR227307, OR227312, OR227313	NR_028148.1
		2	*Methanobacterium formicicum* L21-2	99.58–99.79	OR227308, OR227311	KX344121.1
		2	*Methanosarcina vacuolata* Z-761	98.74–98.98	OR227309, OR227310	CP009520.1
	S1	2	*Methanosarcina vacuolata* Z-761	99.58–99.69	OR227330, OR227331	CP009520.1
3	D3	1	*Methanoculleus sediminis* S3Fa	99.8	OR227314	NR_136474.1
		3	*Methanobacterium formicicum* L21-2	99.69–99.9	OR227317-OR227319	KX344121.1
4	S4	1	*Methanolobus chelungpuianus* St545Mb	99.58	OR227335	NR_177296.1
5	D5	5	*Methanocalculus pumilus* MHT-1	98.86–99.59	OR227320-OR227323, OR227325	NR_028148.1
		1	*Methanoculleus* sp. SLH121	97.04	OR227324	KC893306.1
	S5	2	*Methanosarcina mazei* zm-15	99.71–99.9	OR227338, OR227344	CP042908.1
		4	*Methanofollis liminatans* DSM 4140	99.04–99.69	OR227339-OR227341, OR227351	NR_028254.1
		7	*Methanomicrobium antiquum* DSM 21220	99.38–99.69	OR227342, OR227343, OR227345, OR227346, OR227348-OR227350	CP091092.1
		1	*Methanocalculus pumilus* MHT-1	99.07	OR227347	NR_028148.1
6	D6	2	*Methanolobus* sp. 17PMc2	99.18–99.27	OR227326, OR227327	LC183853.1
	S6	2	*Methanobacterium subterraneum* A8p	99.69–99.9	OR227355, OR227356	CP017768.1
		1	*Methanoculleus marisnigri* JR1	99.49	OR227360	NR_074174.1
		1	*Methanobacterium formicicum* L21-2	99.38	OR227361	KX344121.1
7	D7	1	*Methanothermobacter thermautotrophicus* Delta H	99.69	OR227328	CP064324.1

Methanogens were identified in nearly all samples, either through cultivation and pure colonies isolation or by detecting the autofluorescence of cells in the inoculated medium. No viable methanogens were detected in samples from Object 2, in the deep sample or in the sample from riser pipes. This deep sample was extremely poor in microorganisms and there was almost no growth overall in the serum bottles. A variety of microorganisms grew in the sample from the riser pipes, but no autofluorescence was observed in either temperature variant (37°C and 55°C) during prolonged cultivation. No autofluorescence was observed in the sample R7 either, although the sample D7 was positive for methanogens.

Sample D7 had a uniform morphology of growing methanogens, which were identified as *Methanothermobacter thermautotrophicus* with 99.69% sequence identity based on the sequenced gene for 16S rRNA. The rod-shaped cells forming characteristic long chains were observed quickly after inoculation of the media with the sample. Interestingly, the samples that initially appeared pure were always contaminated with the thermophilic bacterium *Pseudothermotoga elfii* (99.74%) (OR227329), which is common in similar environments. The cells of this bacterium were seen in culture after prolonged cultivation (7 days) and typical purification methods were not successful in obtaining pure culture of the methanogen. The methanogen did not grow in the presence of antibiotics, and newly picked up colonies from repeated agar dilution series did not grow in the absence of the bacterium. *Pseudothermotoga elfii* and *Methanothermobacter thermautotrophicus* formed a biofilm-like structure, which could be seen microscopically (Supplementary Figure S9).

The cultured methanogens usually corresponded with the Illumina sequencing results (Table 2). For example, from sample D1, which had 42% archaeal sequences belonging to the genus *Methanocalculus* and 57% to the family *Methanobacteriaceae* in the Illumina sequencing results, the two species – *Methanocalculus pumilus* and *Methanobacterium formicicum* were actually isolated, with an additional *Methanosarcina* isolate. In sample D5, the predominant methanogen according to sequencing was *Methanocalculus* sp., which was isolated, cultivated and identified as *Methanocalculus pumilus*. Interestingly, it was also possible to isolate *Methanoculleus* sp. which, according to the sequencing results, represents only 0.4% of the archaeal sequences. This shows the importance of cultivation and promoting the growth of some species that might be lost in the data due to their low abundance.

On the other hand, in some cases, the fast-growing and less nutrient-demanding methanogens predominated, as shown by the Illumina sequencing results. Mainly hydrogenotrophic methanogens were isolated, although 16S rRNA sequencing shows a high abundance of methylotrophic species. The species “*Candidatus* Methanoplasma” was not isolated despite its high prevalence in some samples. The methylotrophic *Methanosarcina* sp. or hydrogenotrophic *Methanofollis* sp. always dominated the enriched cultures and were successfully isolated in pure cultures.

#### 3.4.2 Bacteria

The focus of the bacterial community was on SRB. Therefore, the medium for SRB was inoculated and the production of H_2_S was observed by the reaction of Mohr salt and H_2_S. Sulfide production was confirmed by black staining the medium in six samples from four objects. The positive samples were repeatedly reinoculated to the sterile fresh media to obtain a morphologically monotonous SRB community. After final purification by serial dilution on agar plates, the picked colonies were cultivated, DNA isolated and 16S rDNA sequenced. The sequences obtained were identified with a high percentage of identity (>99.3%) as *Desulfovibrio desulfuricans* and *Desulfovibrio alaskensis* (Table 3). Repeated attempts to isolate SRB from two other positive samples, D1 and D5 were unsuccessful. This could be caused by either a higher nutrients requirement, syntrophic relationship between SRB and other bacteria providing SRB with necessary nutrients, a higher sensitivity to oxygen during manipulation processes, or that the sulfide produced was of other origin, e.g., enteric bacteria. It is likely that conditions were not favorable for these SRB, because Illumina sequencing results showed that SRB were present in almost all samples. The unsuccessful cultivation in these cases could be due to a lack of essential nutrients, incorrectly chosen cultivation conditions, or the insufficient volume of inoculum. The medium could also be too favorable for fast-growing *Desulfovibrio* sp., which could overgrow other SRB species in the samples.

**Table 3:** Production of H_2_S from samples during the cultivation and isolated and identified species of sulfate-reducing bacteria from the H_2_S positive samples.

Object	Sample	Sulfate [mg/l]	Production of H_2_S confirmed by cultivation	Closest culturable representative	BLAST identity percentage [%]	Accession number	Reference accession number
1	S1	N.M.	YES	*Desulfovibrio desulfuricans* DSM 233	99.34–99.47	OR227332-OR227334	OQ608761.1
	D1	1.91	YES	–	–	–	–
2	S2	N.M.	NO	–	–	–	–
	D2	3	NO	–	–	–	–
3	D3	2	NO	–	–	–	–
4	S4	N.M.·	YES	*Desulfovibrio alaskensis* HEB223	99.6–99.73	OR227336, OR227337	DQ867001.1
	D4	20.6*	NO	–	–	–	–
5	S5	N.M.·	YES	*Desulfovibrio alaskensis* G20	99.87	OR227352-OR227354	CP000112.1
	D5	57.2**	YES	–	–	–	–
6	S6	N.M.·	YES	*Desulfovibrio desulfuricans* DSM 233	99.47–99.73	OR227357-OR227359	OQ608761.1
	D6	2	NO	–	–	–	–
7	R7	N.M.·	NO	–	–	–	–
	D7	3	NO	–	–	–	–

N.M. – not measured.

*data measured in 2011.

*data measured in 1992.

Positive sulfate reduction was proved in samples from surface technology at UGS with mesophilic temperature in the reservoir. Deep water samples positive for sulfide production originated only from Object 1 and Object 5, but without cultivated representatives. The presence of SRB in surface technology could be closely related to corrosion of metal materials at UGS.

In addition to SRB cultivation, bacterial isolates were also obtained and further identified along the process of isolation methanogenic archaea. The isolation of *Pseudothermotoga elfii* from sample D7 has already been mentioned (3.4.1), but other representatives of the phylum *Thermotogota* were also present in a number of sequenced samples, and some of them were further identified. In sample D3, *Oceanotoga* sp. (OR227315, OR227316) was isolated from the sample with the closest relative *Oceanotoga* sp. DSM 15011 (99.58–99.73%).

The sample from riser pipes was the most diverse sample of all with the predominance of *Pseudomonas* sp., accounting for 80% of all 16S rRNA sequences. This representative was isolated and identified as *Stutzerimonas stutzeri* (formerly *Pseudomonas stutzeri*) (Gomila et al., 2022) (99.59%) (OR260284). *S. stutzeri* is of interest due to its characteristics, as it is capable of degrading polyethylene glycol to ethylene glycol (Lalucat et al., 2006). This is in absolute agreement with the environment from which it was isolated, as polyethylene glycol is used in a gas mining technologies for gas dehydration. The technical fluids serve as a growth substrate for *S. stutzeri.*

### 3.5 Global terrestrial subsurface microbiome comparison

Due to the absence of pure UGS data that would describe microbial composition in operating subsurface systems and their dependence on conditions and UGS type, the data collection included deep subsurface environments, such as boreholes or subsurface mines, oil fields and aquifers (Table 4; Supplementary File 3). The projects that used one primer set for both domains were analyzed separately as the Set 1. In addition, the *Bacteria* and *Archaea* domains included data from projects that had used set of primers only for the specific domain (Set 2 and Set 3, respectively).

**Table 4:** Preview of references, UGS types and BioProject numbers used for processing and analysis of subsurface data.

References	Sample type	Country	BioProject number
Buriánková et al. (2022)	Aquifer and Depleted field	Czech Republic	PRJNA759841
Frank et al. (2016)	Aquifer	Russia	PRJNA349120
Matsushita et al. (2018)	Aquifer	Japan	PRJDB5276
Nazina et al. (2023)	Aquifer	Russia	PRJNA724815
Osburn et al. (2014)	Borehole	USA	PRJNA262938
Ranchou-Peyruse et al. (2021)	Aquifer	France	PRJNA739070
Schwab et al. (2022)	Salt cavern	Germany	PRJEB49822
Vigneron et al. (2017a)	Aquifer	USA	PRJNA325306
Vigneron et al. (2017b)	Oil field	Denmark	PRJNA348365
Zhang et al. (2019)	Borehole	USA	PRJEB35125
Dohrmann and Krüger (2023)	Gas field	Germany	PRJEB51255
Dutta et al. (2018)	Borehole	India	PRJNA389253
Purkamo et al. (2018)	Aquifer	Finland	PRJEB25908
Magnabosco et al. (2014)	Borehole	South Africa	PRJNA263371
Rempfert et al. (2017)	Aquifer	Oman	PRJNA352492
Bian et al. (2023)	Oil field	China	PRJNA884886
Gao et al. (2019)	Oil field	China	PRJNA489604
Sokolova et al. (2021)	Oil field	Kazakhstan	PRJNA749317

**Figure 4:**
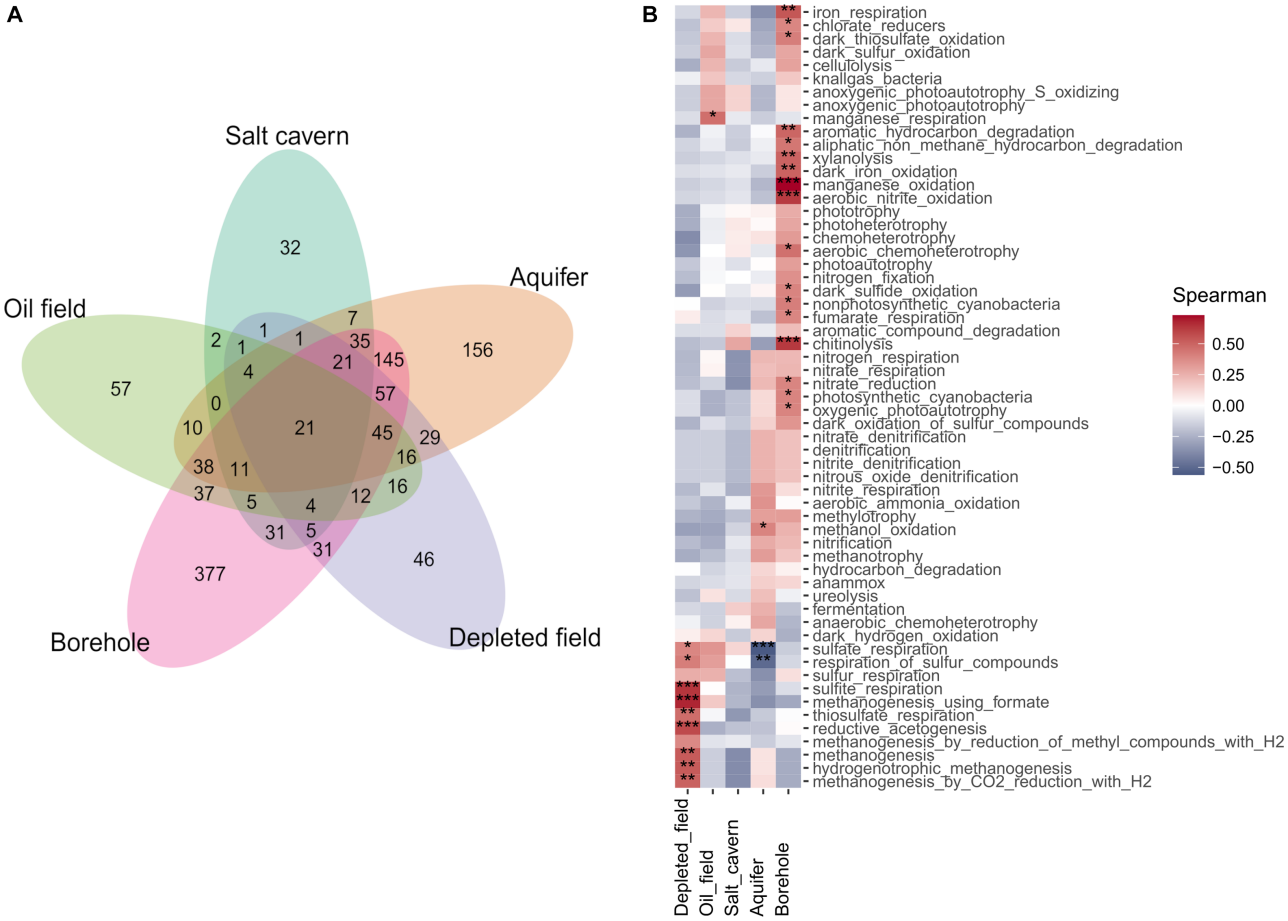
**(A)** Venn diagram of Set 1 of unique taxa belonging to different types of samples portrayed in petal plot. **(B)** Correlation constructed heatmap of Set 1 different samples type and predicted metabolic pathways predicted by FAPROTAX, using Spearman correlation method (*p < 0.05, **p < 0.01, ***p < 0.001).

**Figure 5:**
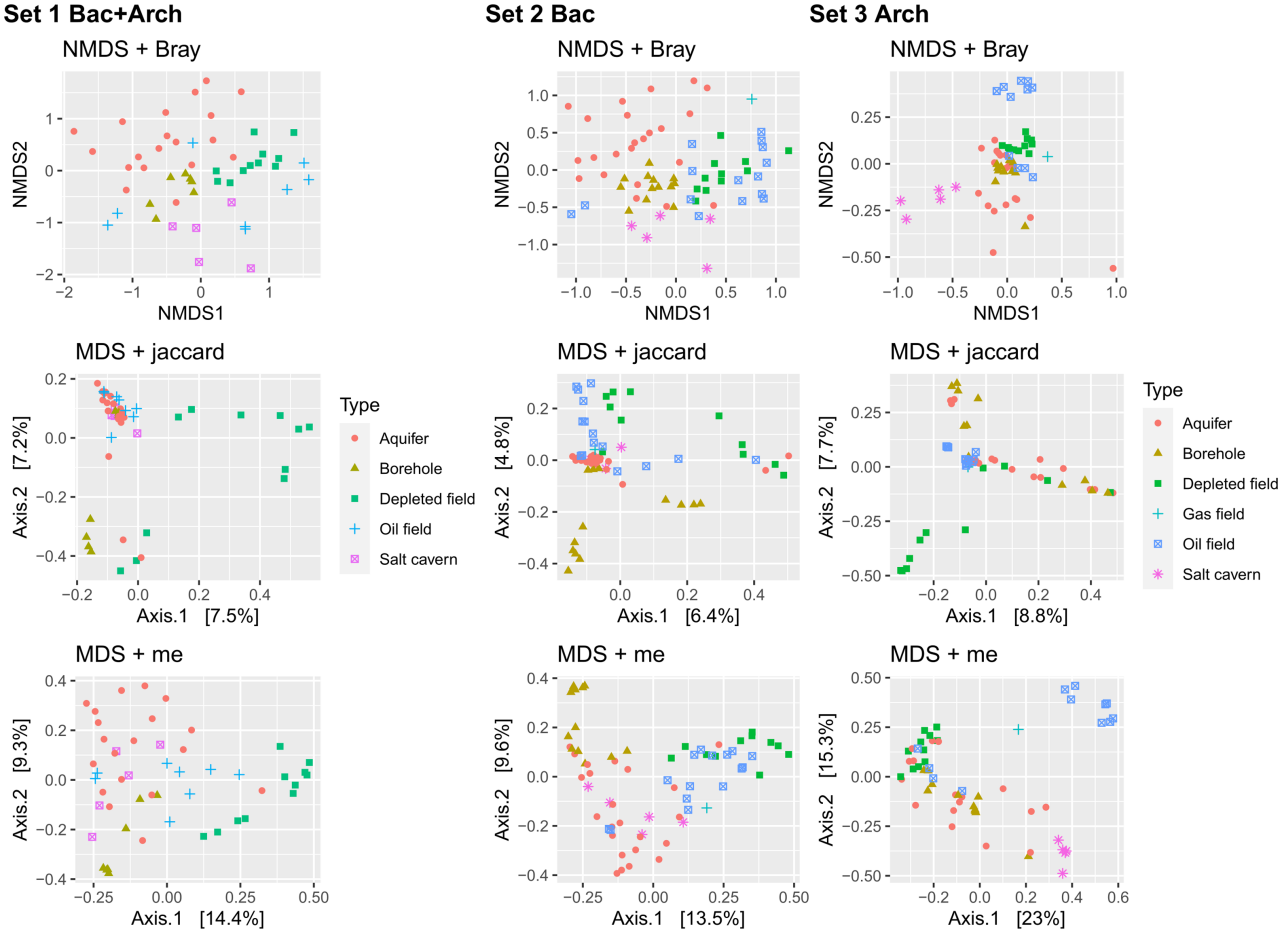
Non-metric multidimensional scaling (NMDS) component plot using Bray-Curtis distance method and multidimensional scaling (MDS) component plot using Jaccard method and me method, both showing the processed 16S rRNA data of samples from underground environment and subdivided by sample type. The Set 1 contained data from domain *Bacteria* and *Archaea*, the Set 2 contained data only from domain *Bacteria* and Set 3 contained data only from domain *Archaea*.

Performing the PERMANOVA test on the datasets showed a significant difference between sample types (*p* < 0.05) in all sets and distance methods used. Analysis of the data using the non-metric multidimensional scaling method with Bray-Curtis distance (Figure 4) showed only slight clustering of samples of the same type in the case of Set 1 and Set 2. The patterns of clustering showed aquifer type samples, salt caverns and also boreholes. Set 3, which contained only filtered sequences of *Archaea* domain, showed a different pattern with clusters from oil fields and salt caverns, probably due to the extreme conditions in these environments (halophilic and thermophilic), which led to a differentiation of archaeal communities specific only to the type or origin of the sample. Similar trends are found also in multidimensional scaling using Jaccard and me method, for both depleted field samples and borehole distant from other samples. The aquifer cluster often transitions slowly into the borehole in sets, indicating similarity between these two environments, as seen in the Venn diagram, where the aquifers and boreholes share the most common unique taxa, 145 (Figure 5A). Only 21 taxa were shared by all sample types. The shared taxa were comprised of common bacterial genera, such as *Staphylococcus, Streptococcus, Bacillus, Acinetobacter, Pseudomonas, Sphingomonas* and others (Supplementary File 3). The high abundance of order *Pseudomonadales*, *Burkholderiales* and *Rhizobiales* can also be seen in Supplementary Figure S10. In Set 3 with only archaea, no shared taxa were found between samples, although the most abundant order in all samples is *Methanosarcinales*, followed by *Methanobacteriales* (Supplementary Figure S11).

Spearman correlation between predicted metabolic pathways and sample type shows a significant correlation (*p* < 0.05) between the different methanogenesis pathways and the depleted oil field samples (Figure 5B). The strong methanogenesis trait could be explained by the previously described 16S rRNA sequencing results and the unexpectedly high proportion of archaea. Samples from boreholes are the most diverse, depending on the type of borehole from which they were collected and the geochemical composition of the bedrock. Due to the presence of various chemical elements in the bedrock, the metabolism of the microorganisms was adapted to the conditions, resulting in a significant relationship between different alternative electron acceptor pathways and borehole type. There is a significant correlation between the borehole samples and iron respiration, thiosulphate and sulfur respiration, fumarate respiration and oxidation (*p* < 0.05). Interestingly, there is a negative correlation (*p* < 0.001) between aquifer type and sulfate, which could be due to the low amounts of sulfate in the sequenced aquifers. When analyzing the different environmental conditions and the predicted correlation of microbial metabolic pathways, there is a significant (*p* < 0.05) correlation between increasing pH and metabolic pathways such as methanogenesis, sulfite and thiosulphate respiration, and reductive acetogenesis (Supplementary Figure S12). This is interesting as these pathways are associated with hydrogen utilization. Some of the SRBs are able to reduce other sulfur compounds such as thiosulphate or sulfite using hydrogen as an electron donor. The produced compound, hydrogen sulfide, causes decrease of pH. Similarly, acetogens produce acetic acid from hydrogen and carbon dioxide. This is contrast to the fact that the correlation is positive, which means that higher a pH in the environment is connected with a higher abundance of this group of microorganisms (Supplementary Figure S12). This phenomenon could be explained by looking more closely at the correlations between the environment and each genus of Set 2 and Set 3 (Supplementary Figures S13A, S14A). There is number of SRBs (*Dethiobacter, Desulfatitalea*, *Desulfonatronum*, *Desulfatiglans*) with positive correlation (*p* < 0.05) to increasing pH. This group of SRBs consists of alkaliphilic species (Higashioka et al., 2013; Suzuki et al., 2014; Khomyakova et al., 2023), which cause the correlation to pH. Statistically significant negative correlation of SRB and pH was found in genus *Desulfovermiculus*, which also has positive correlation with salt cavern type. The representatives of this genus grow at neutral pH but are halophilic (Belyakova et al., 2006; Jakobsen et al., 2006). In archaeal Set 3, the positive statistically significant correlation (*p* < 0.05) between increasing pH and genera is found in the genus *Methanocalculus*, which includes a number of halotolerant species with pH tolerance (Ollivier et al., 1998; Lai et al., 2002; Zhilina et al., 2013), and not significantly in the genus *Methanobacterium*, which is also endowed with pH tolerance (Worakit et al., 1986; Kotelnikova et al., 1998; Shlimon, 2004; Mori and Harayama, 2011). Genus *Methanothermobacter* and *Methermicoccus* have a significant correlation (*p* < 0.05) with depth and temperature and also with oil field, which is expected due to their optimal conditions and conditions in deep wells (Supplementary Figure S14A). Salt caverns also have an expected positive correlation (*p* < 0.001) with halophilic genera, such as *Halanaeroarchaeum*, *Methanohalophilus*, *Halodesulfurarchaeum*, *Halorhabdus*, *Haloarcula*, *Halapricum*, *Natronomonas* and “*Candidatus* Haloredivivus” (Supplementary Figure S14B).

## 4 Discussion

Subsurface environments are still quite unexplored areas that have become increasingly important in recent years, especially with the growing interest in hydrogen storage or subsurface methanation. There are over 250 UGSs in Europe and almost 700 UGSs in operation worldwide (IGU, 2023), but less is known about the microbial communities there and possible interactions between the microorganisms and the stored gas that affect the potential for hydrogen storage.

In our study, microbial communities in UGS of depleted gas fields were investigated. The main focus was on methanogenic archaea and subsequently SRB as potential hydrogen consumers for possible hydrogen supply. Samples were collected from various objects with different depth and temperature, to observe the influence of environmental conditions on microbial community composition.

The combination of molecular biology methods and cultivation allowed us to gain insight into communities, their viability, close identification of isolated species and also their response to the hydrogen addition. The isolated species were mostly consistent with the results of 16S rRNA sequencing. The isolated species belonged to the orders *Methanomicrobiales*, *Methanosarcinales* and *Methanobacteriales*. The species obtained from deep water samples were mostly hydrogenotrophic, which is related to the presence of syntrophic species of phylum *Thermotogota* and *Synergystota*, as well as *Bacillota*. Some of the representatives are producers of hydrogen, which serves as substrate for hydrogenotrophic methanogens. In contrast, methylotrophic methanogens, namely the order *Methanosarcinales* and *Methanomassiliicoccales* dominated in separators. Their relatively high abundance could be caused by presence of methanol as technical fluid in natural gas upgrading process. Methanol is the main substrate used by methanogens but is toxic to many bacteria and other archaea. This high amount of “*Candidatus* Methanoplasma” and the high archaeal ratio was very unexpected and is not commonly observed in similar environments.

In sample D2 with a depth of 1,900 m, no methanogens could be cultivated. We were also unable to recover enough DNA. The temperature and pressure shock to which the microorganisms were subjected was much higher compared to the other samples during the sampling. Obtaining methanogens through cultivation methods serves as a confirmation of the existence of viable methanogens within the underground gas storage (UGS) environment. This finding holds significant importance, as it aligns with the results obtained through Next-Generation Sequencing (NGS) and has critical implications for planned hydrogen storage or *in situ* methanation efforts. Additionally, cultivation techniques have the unique capability of identifying species that might constitute a minority in the initial samples, species that might otherwise be overlooked during data analysis, sample processing, or DNA isolation. Some of these species are responsive to hydrogen supplementation and could be regarded as the ones most influenced by the introduction of hydrogen, a characteristic not discernible solely from NGS data. Relying exclusively on NGS data to assess the presence of methanogens could potentially lead to unforeseen complications in hydrogen storage applications. There is potential risk in the change of the proportion of methanogens after addition of hydrogen to the environment, and dominance of methanogenic species, that are usually represented in the microbiome in small amounts. Cultivation showed that their presence is also important for further studies and hydrogen storage, as methanogens can become predominant in the microbiome after hydrogen and CO_2_ stimulation (Vítězová et al., 2023).

Compared to our prior investigations into the underground gas storage (UGS) environment (Buriánková et al., 2022; Vítězová et al., 2023), the UGS community in this specific location differed significantly from previously sequenced UGS wells in the Vienna Basin in the Czech Republic. The distinct separation between wells and the variations in long-term operation protocols among them yielded unexpectedly diverse microbiomes. This underscores that it’s not just the geographical location but also the unique characteristics of individual wells that play a crucial role. The geological structure of the UGS contributes to variations in several key parameters, such as temperature (which increases with depth) and the presence of carbon in the form of hydrocarbons, among others, shaping the distinct microbiome of the sampled UGS site. These differences are consequential in the planning of methanation or hydrogen storage and are not only notable within our research but also extend to the analysis of microbiomes in similar UGS environments.

The comparison and analysis of different terrestrial subsurface samples shows variability in metabolic pathways and microbial communities. Although the dataset is limited, which could strongly influence the results, interesting features could be seen, including similarities between different environments or correlation with different conditions. The exploration of subsurface environment is a key element for further application.

The discovery of similarities among the sample types is an intriguing observation with potential implications for further analyses and predictions, particularly when focusing on a specific type of underground gas storage (UGS) that piques our interest. As illustrated in Figure 4, the differentiation between various UGS types is reflected in the microbiome structure, which exhibits notably distinct patterns when comparing Set 2 with the Bacteria domain and Set 3 with the Archaea domain. Given that bacteria constitute the majority of the microbiome, it’s unsurprising to find that the plot displaying Set 1 with all domains closely resembles Set 2, although it’s enlightening because Set 2 contains a more extensive dataset than Set 1. However, our primary interest lies in methanogens, particularly for hydrogen (H_2_) storage purposes. In this context, Set 3 emerges as the most crucial dataset due to its distinctive pattern, which clusters together more subsurface-type samples. This clustering could facilitate the modeling process for overall H_2_ storage. The selection of key representatives of different UGS types based on the results following our analysis and incorporating their physiological characteristics could contribute to the broader mathematical modeling of methanation or H_2_ storage. To enhance the specificity of our modeling and increase accuracy, we have presented detailed data concerning the behavior of the subsurface environment and identified common features that can be employed for future predictions.

In this study, no negative correlation was found between methanogenesis and specific environments, so there is a probability, that methanogenesis could occur during H_2_ storage in all environments. However, salt caverns showed non-significant negative correlation with methanogens in this limited dataset and also a different microbial and archaeal profile, which might support the idea of focusing on salt caverns for H_2_ storage. Of all the methanogens, only the order *Methanosarcinales* was present, whose metabolism is mostly methylotrophic and acetoclastic, and whose hydrogen utilization depends on the species present, which have to be assessed individually through cultivation techniques. The species can overcome salinity up to 1.5 M, however, the growth rate decreases under these conditions (Maestrojuan and Boone, 1991). Based on the available data, no halophilic hydrogenotrophic methanogens were observed, but this does not mean that they could not occur in other salt caverns. Some of the hydrogenotrophic methanogens are able to grow in the presence of 3 M Na^+^ concentration (Ollivier et al., 1998; Zhilina et al., 2013), however, their presence in the salt cavern microbiome remains to be confirmed.

Nevertheless, the absence of methanogens is associated with the presence of sulfate-reducers, which pose an even greater problem as they are associated with hydrogen consumption and also corrosion (Dinh et al., 2004; Enning et al., 2012). Sulfate levels in aquifers were low and showed a negative correlation with SRB, which is consistent with the fact that aquifers are also often considered as potential H_2_ storages. Aquifers were often colonized by the order *Methanobacteriales* (Supplementary Figure S11), which are hydrogenotrophic, so it depends on the specific conditions of the aquifer, salinity and temperature to predict the methanogenesis rate under such conditions during H_2_ storage. Samples from different temperatures up to 100°C showed the presence of methanogens in all environments. The thermophilic methanogens often have faster growth rate and thus a higher rate of methanogenesis (Stetter et al., 1981; Jones et al., 1983; Burggraf et al., 1990; Kurr et al., 1991; Mauerhofer et al., 2021). Since there are hydrogenotrophic methanogens that colonize environments with temperatures up to 120°C (Kurr et al., 1991), it is more difficult to find UGS that fit into these extreme conditions to exclude the presence of methanogens. In the case of low-temperature UGS, the pressure inside compensates for the slower growth rate of mesophilic methanogens.

The rich microbial community inhabiting the environment serves as a significant yet underexplored facet of hydrogen (H_2_) storage. This microbial diversity varies across different samples, necessitating the assessment of each prospective underground gas storage (UGS) site for H_2_ storage potential. This study highlights commonalities among distinct subsurface sample types and identifies representative species within each environment. This information offers valuable insights into predicting the microbial composition in analogous environments, focusing on potential microorganisms within UGS sites, and forecasting their metabolic pathways. Anticipating the presence of specific microbial species holds the potential to inform the development of mathematical models that incorporate biological aspects into H_2_ storage or methanation models.

Modeling methanation within underground gas storage (UGS) sites using specific species under varying conditions can yield crucial insights for Power-to-Gas technology and conversion rates. As the development of the hydrogen (H_2_) economy continues, *in-situ* methanation might become technologically viable sooner than the utilization of pure green H_2_. Conversely, the storage of H_2_ raises questions about the behavior of methanogens in the absence of CO_2_ supplementation, particularly when their carbon source relies on accessible carbonates in the subsurface environment (Bo et al., 2021; Dopffel et al., 2021; Aftab et al., 2022). This presents a challenge for model development and is a pivotal factor in the context of hydrogen (H_2_) storage within underground gas storage (UGS) facilities and also a limiting factor for methanogenesis in the UGS during H_2_ storage. Nevertheless, the substantial volume of UGS and the subsurface water flow may mitigate CO_2_ limitations during storage. Recent laboratory simulations involving the introduction of pure H_2_ into UGS water have shed light on the activities of sulfate-reducing bacteria (SRB) and methanogens (Haddad et al., 2022; Dohrmann and Krüger, 2023). In one case, SRBs outcompeted methanogens (Dohrmann and Krüger, 2023), while in another (Haddad et al., 2022), hydrogen consumption led to a significant increase in the methanogen population. Hydrogen consumption ceased after an 80 day incubation period during which no detectable source of CO_2_ was observed. Hence, the pivotal query pertains to the feasibility and mechanisms for averting methanogenesis during the storage of hydrogen in extensive environments like underground gas storage facilities (UGSs). Regrettably, this question remains unresolved so far.

## 5 Conclusion

Our findings suggest the presence of diverse and live microorganisms including the hydrogen-consuming methanogens and sulfate reducers in underground gas storage facilities. Although the results are descriptive and we do not know how active these microorganisms are *in situ*, we could still postulate their potential impacts on underground hydrogen gas storage based on the well-established physiology of methanogens and sulfate reducers. Since methanogens specialize in converting hydrogen into methane, they do not benefit the hydrogen storage, as they could contribute to losses of the hydrogen gas during long-term storage. However, suitable support of methanogenesis in form of hydrogen and CO_2_ gas combination could lead to production of green methane, which can be immediately used for injection into gas grid. This is because methane is long established energy carrier that has a higher energy density and is easier to store than hydrogen. Conversely, sulfate reducers convert hydrogen into corrosive and toxic hydrogen sulfide, making them detrimental to hydrogen storage. A better understanding of these and other hydrogen-consuming microorganisms in underground gas storage facilities will help us develop safer and more efficient ways for long term hydrogen storage. In this context, it will be essential to evaluate whether underground gas storage can serve as fermenter for methane production in Power-to-Gas technology or as repositories for long term hydrogen storage, where methanation is undesirable. A complex analysis of 16S rRNA gene of the existing data offers a comprehensive perspective that can be harnessed in the creation of mathematical models that integrate biological components into models for hydrogen storage and methanation. However, it’s crucial to acknowledge the limitations of our study, notably the restricted dataset of samples available in the case of certain subsurface types, such as salt caverns, due to their exclusivity. Further investigations of these samples are essential to enhance our comprehension of their composition and characteristics.

## Data availability statement

The data have been deposited with links to BioProject accession number PRJNA1014364 in the NCBI BioProject database (https://www.ncbi.nlm.nih.gov/bioproject/).

## Author contributions

NH: Conceptualization, Formal analysis, Investigation, Methodology, Visualization, Writing – original draft, Writing – review & editing. MV: Conceptualization, Formal analysis, Methodology, Resources, Validation, Writing – original draft, Writing – review & editing. TV: Conceptualization, Formal analysis, Methodology, Writing – original draft, Writing – review & editing. IK: Writing – review & editing. EK: Investigation, Writing – review & editing. DN: Investigation, Validation, Visualization, Writing – review & editing. JL: Investigation, Validation, Visualization, Writing – review & editing. RZ: Conceptualization, Methodology, Writing – review & editing.

## References

[ref1] Aftab A. Hassanpouryouzband A. Xie Q. Machuca L. L. Sarmadivaleh M. (2022). Toward a fundamental understanding of geological hydrogen storage. Ind. Eng. Chem. Res. 61, 3233–3253. doi: 10.1021/acs.iecr.1c04380

[ref2] Apprill A. McNally S. Parsons R. Weber L. (2015). Minor revision to V4 region SSU rRNA 806R gene primer greatly increases detection of SAR11 bacterioplankton. Aquat. Microb. Ecol. 75, 129–137. doi: 10.3354/ame01753

[ref3] Belyakova E. V. Rozanova E. P. Borzenkov I. A. Tourova T. P. Pusheva M. A. Lysenko A. M.. (2006). The new facultatively chemolithoautotrophic, moderately halophilic, sulfate-reducing bacterium *Desulfovermiculus halophilus* gen. Nov., sp. Nov., isolated from an oil field. Microbiology 75, 161–171. doi: 10.1134/S0026261706020093 16758868

[ref4] Bian Z. Chen Y. Zhi Z. Wei L. Wu H. Wu Y. (2023). Comparison of microbial community structures between oil and water phases in a low-permeability reservoir after water flooding. Energy Rep. 9, 1054–1061. doi: 10.1016/j.egyr.2022.12.026

[ref5] Bo Z. Zeng L. Chen Y. Xie Q. (2021). Geochemical reactions-induced hydrogen loss during underground hydrogen storage in sandstone reservoirs. Int. J. Hydrog. Energy 46, 19998–20009. doi: 10.1016/j.ijhydene.2021.03.116

[ref6] Burggraf S. Fricke H. Neuner A. Kristjansson J. Rouvier P. Mandelco L.. (1990). *Methanococcus igneus* sp. Nov., a novel hyperthermophilic methanogen from a shallow submarine hydrothermal system. Syst. Appl. Microbiol. 13, 263–269. doi: 10.1016/S0723-2020(11)80197-9 11538305

[ref7] Buriánková I. Molíková A. Vítězová M. Onderka V. Vítěz T. Urbanová I.. (2022). Microbial communities in underground gas reservoirs offer promising biotechnological potential. Fermentation 8, 1–16. doi: 10.3390/fermentation8060251

[ref8] Buzek F. Onderka V. Vancura P. Wolf I. (1994). Carbon isotope study of methane production in a town gas storage reservoir. Fuel 73, 747–752. doi: 10.1016/0016-2361(94)90019-1

[ref9] Casamayor E. O. Massana R. Benlloch S. Ovreas L. Diez B. Goddard V. J.. (2002). Changes in archaeal, bacterial and eukaryal assemblages along a salinity gradient by comparison of genetic fingerprinting methods in a multipond solar saltern. Environ. Microbiol. 4, 338–348. doi: 10.1046/j.1462-2920.2002.00297.x 12071979

[ref10] Cheng L. Qiu T.-L. Li X. Wang W.-D. Deng Y. Yin X.-B.. (2008). Isolation and characterization of *Methanoculleus receptaculi* sp. Nov. from Shengli oil field, China. FEMS Microbiol. Lett. 285, 65–71. doi: 10.1111/j.1574-6968.2008.01212.x 18557787

[ref11] Cristello J. B. Yang J. M. Hugo R. Lee Y. Park S. S. (2023). Feasibility analysis of blending hydrogen into natural gas networks. Int. J. Hydrog. Energy 48, 17605–17629. doi: 10.1016/j.ijhydene.2023.01.156

[ref12] Dinh H. T. Kuever J. Mussmann M. Hassel A. W. Stratmann M. Widdel F. (2004). Iron corrosion by novel anaerobic microorganisms. Nature 427, 829–832. doi: 10.1038/nature02321 14985759

[ref13] Dohrmann A. B. Krüger M. (2023). Microbial H_2_ consumption by a formation fluid from a natural gas field at high-pressure conditions relevant for underground H_2_ storage. Environ. Sci. Technol. 57, 1092–1102. doi: 10.1021/acs.est.2c07303 36599497

[ref14] Dopffel N. Jansen S. Gerritse J. (2021). Microbial side effects of underground hydrogen storage – knowledge gaps, risks and opportunities for successful implementation. Int. J. Hydrog. Energy 46, 8594–8606. doi: 10.1016/j.ijhydene.2020.12.058

[ref15] Douglas G. M. Maffei V. J. Zaneveld J. R. Yurgel S. N. Brown J. R. Taylor C. M.. (2020). PICRUSt2 for prediction of metagenome functions. Nat. Biotechnol. 38, 685–688. doi: 10.1038/s41587-020-0548-6 32483366 PMC7365738

[ref16] Dutta A. Dutta Gupta S. Gupta A. Sarkar J. Roy S. Mukherjee A.. (2018). Exploration of deep terrestrial subsurface microbiome in late cretaceous Deccan traps and underlying Archean basement, India. Sci. Rep. 8:17459. doi: 10.1038/s41598-018-35940-0, PMID: 30498254 PMC6265293

[ref17] Eder W. Ludwig W. Huber R. (1999). Novel 16S rRNA gene sequences retrieved from highly saline brine sediments of Kebrit Deep, Red Sea. Arch. Microbiol. 172, 213–218. doi: 10.1007/s002030050762 10525737

[ref18] Enning D. Venzlaff H. Garrelfs J. Dinh H. T. Meyer V. Mayrhofer K.. (2012). Marine sulfate-reducing bacteria cause serious corrosion of iron under electroconductive biogenic mineral crust. Environ. Microbiol. 14, 1772–1787. doi: 10.1111/j.1462-2920.2012.02778.x 22616633 PMC3429863

[ref19] Falzolgher F. Altieri G. (2005). “Underground storage of natural gas” in Encyclopaedia of hydrocarbons: Exploration, production and transport. eds. In P. F. Barnaba G. B. Mazzei R. (Rome: ENI), 879–910.

[ref20] Frank Y. A. Kadnikov V. V. Gavrilov S. N. Banks D. Gerasimchuk A. L. Podosokorskaya O. A.. (2016). Stable and variable parts of microbial community in Siberian deep subsurface thermal aquifer system revealed in a long-term monitoring study. Front. Microbiol. 7:2101. doi: 10.3389/fmicb.2016.02101 28082967 PMC5187383

[ref21] Galyas A. B. Kis L. Tihanyi L. Szunyog I. Vadaszi M. Koncz A. (2023). Effect of hydrogen blending on the energy capacity of natural gas transmission networks. Int. J. Hydrog. Energy 48, 14795–14807. doi: 10.1016/j.ijhydene.2022.12.198

[ref22] Gao P. Li Y. Tan L. Guo F. Ma T. (2019). Composition of bacterial and archaeal communities in an alkali-surfactant-polyacrylamide-flooded oil reservoir and the responses of microcosms to nutrients. Front. Microbiol. 10:2197. doi: 10.3389/fmicb.2019.02197 31611855 PMC6777151

[ref23] Gay P. Chalumeau H. Steinmetz M. (1983). Chromosomal localization of gut, fruC, and pfk mutations affecting genes involved in *Bacillus subtilis* D-glucitol catabolism. J. Bacteriol. 153, 1133–1137. doi: 10.1128/jb.153.3.1133-1137.1983 6402486 PMC221755

[ref24] GIE (2021). Gas Infrastructure Europe. Available at: https://www.gie.eu/

[ref25] Gniese C. Bombach P. Rakoczy J. Hoth N. Schlömann M. Richnow H.-H.. (2014). “Relevance of deep-subsurface microbiology for underground gas storage and geothermal energy production” in Geobiotechnology II: energy resources, subsurface technologies, organic pollutants and mining legal principles. eds. Schippers A. Glombitza F. Sand W. (Berlin/Heidelberg: Springer), 95–121. 10.1007/10_2013_257 24311044

[ref26] Godsy E. M. (1980). Isolation of *Methanobacterium bryantii* from a deep aquifer by using a novel broth-antibiotic disk method. Appl. Environ. Microbiol. 39, 1074–1075. doi: 10.1128/AEM.39.5.1074-1075.1980 16345569 PMC291478

[ref27] Gomila M. Mulet M. García-Valdés E. Lalucat J. (2022). Genome-based taxonomy of the genus Stutzerimonas and proposal of *S. frequens* sp. Nov. and *S. degradans* sp. Nov. and emended descriptions of *S. perfectomarina* and *S. chloritidismutans*. Microorganisms 10:1363. doi: 10.3390/microorganisms10071363 35889082 PMC9320692

[ref28] Haddad P. G. Ranchou-Peyruse M. Guignard M. Mura J. Casteran F. Ronjon-Magand L.. (2022). Geological storage of hydrogen in deep aquifers – an experimental multidisciplinary study. Energy Environ. Sci. 15, 3400–3415. doi: 10.1039/D2EE00765G

[ref29] Hanišáková N. Vítězová M. Rittmann S. K.-M. R. (2022). The historical development of cultivation techniques for methanogens and other strict anaerobes and their application in modern microbiology. Microorganisms 10:412. doi: 10.3390/microorganisms10020412 35208865 PMC8879435

[ref30] Heinemann N. Alcalde J. Miocic J. M. Hangx S. J. T. Kallmeyer J. Ostertag-Henning C.. (2021). Enabling large-scale hydrogen storage in porous media – the scientific challenges. Energy Environ. Sci. 14, 853–864. doi: 10.1039/D0EE03536J

[ref31] Higashioka Y. Kojima H. Watanabe M. Fukui M. (2013). Desulfatitalea tepidiphila gen. Nov., sp. Nov., a sulfate-reducing bacterium isolated from tidal flat sediment. Int. J. Syst. Evol. Microbiol. 63, 761–765. doi: 10.1099/ijs.0.043356-0 22581901

[ref32] Hiller H. Reimert R. Marschner F. Renner H.-J. Boll W. Supp E.. (2006). “Gas production. In Wiley-VCH Verlag GmbH & Co. KGaA” in Ullmann’s encyclopedia of industrial chemistry (Weinheim: Wiley-VCH Verlag GmbH & Co. KGaA).

[ref33] Hirano S. Ihara S. Wakai S. Dotsuta Y. Otani K. Kitagaki T.. (2022). Novel Methanobacterium strain induces severe corrosion by retrieving electrons from Fe0 under a freshwater environment. Microorganisms 10:270. doi: 10.3390/microorganisms10020270 35208725 PMC8880523

[ref34] Hogeweg S. Strobel G. Hagemann B. (2022). Benchmark study for the simulation of underground hydrogen storage operations. Comput. Geosci. 26, 1367–1378. doi: 10.1007/s10596-022-10163-5

[ref35] IGU (2023). IGU. Available at:http://ugs.igu.org/index.php/ugs_list/get_list# (Accessed July 10, 2023).

[ref36] Ivanova A. E. Borzenkov I. A. Tarasov A. L. Milekhina E. I. Belyaev S. S. (2007). A microbiological study of an underground gas storage in the process of gas extraction. Microbiology 76, 461–468. doi: 10.1134/S0026261707040121 17974210

[ref37] Jakobsen T. F. Kjeldsen K. U. Ingvorsen K. (2006). *Desulfohalobium utahense* sp. Nov., a moderately halophilic, sulfate-reducing bacterium isolated from great salt Lake. Int. J. Syst. Evol. Microbiol. 56, 2063–2069. doi: 10.1099/ijs.0.64323-0 16957100

[ref38] Jones W. J. Leigh J. A. Mayer F. Woese C. R. Wolfe R. S. (1983). *Methanococcus jannaschii* sp. Nov., an extremely thermophilic methanogen from a submarine hydrothermal vent. Arch. Microbiol. 136, 254–261. doi: 10.1007/BF00425213

[ref39] Kato S. Yumoto I. Kamagata Y. (2015). Isolation of Acetogenic bacteria that induce biocorrosion by utilizing metallic iron as the sole electron donor. Appl. Environ. Microbiol. 81, 67–73. doi: 10.1128/AEM.02767-14 25304512 PMC4272740

[ref40] Khomyakova M. A. Merkel A. Y. Segliuk V. S. Slobodkin A. I. (2023). Desulfatitalea alkaliphila sp. Nov., an alkalipilic sulfate- and arsenate- reducing bacterium isolated from a terrestrial mud volcano. Extremophiles Life Under Extreme Condit. 27:12. doi: 10.1007/s00792-023-01297-0 37178152

[ref41] Kotelnikova S. Macario A. J. L. Pedersen K. (1998). *Methanobacterium subterraneum* sp. Nov., a new alkaliphilic, eurythermic and halotolerant methanogen isolated from deep granitic groundwater. Int. J. Syst. Evol. Microbiol. 48, 357–367. doi: 10.1099/00207713-48-2-357 9731274

[ref42] Kurr M. Huber R. König H. Jannasch H. W. Fricke H. Trincone A.. (1991). *Methanopyrus kandleri*, gen. And sp. Nov. represents a novel group of hyperthermophilic methanogens, growing at 110°C. Arch. Microbiol. 156, 239–247. doi: 10.1007/BF00262992

[ref43] Lai M.-C. Chen S.-C. Shu C.-M. Chiou M.-S. Wang C.-C. Chuang M.-J.. (2002). *Methanocalculus taiwanensis* sp. Nov., isolated from an estuarine environment. Int. J. Syst. Evol. Microbiol. 52, 1799–1806. doi: 10.1099/00207713-52-5-1799 12361289

[ref44] Lalucat J. Bennasar A. Bosch R. García-Valdés E. Palleroni N. J. (2006). Biology of *Pseudomonas stutzeri*. Microbiol. Mol. Biol. Rev. 70, 510–547. doi: 10.1128/MMBR.00047-05 16760312 PMC1489536

[ref45] Li Y. Xu D. Chen C. Li X. Jia R. Zhang D.. (2018). Anaerobic microbiologically influenced corrosion mechanisms interpreted using bioenergetics and bioelectrochemistry: a review. J. Mater. Sci. Technol. 34, 1713–1718. doi: 10.1016/j.jmst.2018.02.023

[ref46] Liang R. Davidova I. Hirano S. Duncan K. E. Suflita J. M. (2019). Community succession in an anaerobic long-chain paraffin-degrading consortium and impact on chemical and electrical microbially influenced iron corrosion. FEMS Microbiol. Ecol. 95:fiz111. doi: 10.1093/femsec/fiz111 31281924

[ref47] Liu C. Cui Y. Li X. Yao M. (2021). microeco: an R package for data mining in microbial community ecology. FEMS Microbiol. Ecol. 97:fiaa255. doi: 10.1093/femsec/fiaa255 33332530

[ref48] Louca S. Parfrey L. W. Doebeli M. (2016). Decoupling function and taxonomy in the global ocean microbiome. Science 353, 1272–1277. doi: 10.1126/science.aaf4507 27634532

[ref49] Maestrojuan G. M. Boone D. R. (1991). Characterization of *Methanosarcina barkeri* MST and 227, *Methanosarcina mazei* S-6T, and *Methanosarcina vacuolata* Z-761T. Int. J. Syst. Bacteriol. 41, 267–274. doi: 10.1099/00207713-41-2-267

[ref50] Magnabosco C. Tekere M. Lau M. C. Y. Linage B. Kuloyo O. Erasmus M.. (2014). Comparisons of the composition and biogeographic distribution of the bacterial communities occupying South African thermal springs with those inhabiting deep subsurface fracture water. Front. Microbiol. 5:679. doi: 10.3389/fmicb.2014.00679 25566203 PMC4269199

[ref51] Matsushita M. Magara K. Sato Y. Shinzato N. Kimura H. (2018). Geochemical and microbiological evidence for microbial methane production in deep aquifers of the cretaceous Accretionary prism. Microbes Environ. 33, 205–213. doi: 10.1264/jsme2.ME17199 29899169 PMC6031385

[ref52] Mauerhofer L.-M. Zwirtmayr S. Pappenreiter P. Bernacchi S. Seifert A. H. Reischl B.. (2021). Hyperthermophilic methanogenic archaea act as high-pressure CH_4_ cell factories. Commun. Biol. 4:289. doi: 10.1038/s42003-021-01828-5 33674723 PMC7935968

[ref53] McMurdie P. J. Holmes S. (2013). phyloseq: an R package for reproducible interactive analysis and graphics of microbiome census data. PLoS One 8:e61217. doi: 10.1371/journal.pone.0061217 23630581 PMC3632530

[ref54] Meda U. S. Bhat N. Pandey A. Subramanya K. N. Lourdu Antony Raj M. A. (2023). Challenges associated with hydrogen storage systems due to the hydrogen embrittlement of high strength steels. Int. J. Hydrog. Energy 48, 17894–17913. doi: 10.1016/j.ijhydene.2023.01.292

[ref55] Molíková A. Vítězová M. Vítěz T. Buriánková I. Huber H. Dengler L.. (2022). Underground gas storage as a promising natural methane bioreactor and reservoir? J. Energy Storage 47:103631. doi: 10.1016/j.est.2021.103631

[ref56] Mori K. Harayama S. (2011). *Methanobacterium petrolearium* sp. Nov. and *Methanobacterium ferruginis* sp. Nov., mesophilic methanogens isolated from salty environments. Int. J. Syst. Evol. Microbiol. 61, 138–143. doi: 10.1099/ijs.0.022723-0 20173004

[ref57] Morozova D. Wandrey M. Alawi M. Zimmer M. Vieth A. Zettlitzer M.. (2010). Monitoring of the microbial community composition in saline aquifers during CO_2_ storage by fluorescence in situ hybridisation. Int. J. Greenhouse Gas Control 4, 981–989. doi: 10.1016/j.ijggc.2009.11.014

[ref58] Morozova D. Zettlitzer M. Let D. Würdemann H. (2011). Monitoring of the microbial community composition in deep subsurface saline aquifers during CO_2_ storage in Ketzin, Germany. Energy Procedia 4, 4362–4370. doi: 10.1016/j.egypro.2011.02.388

[ref59] Nazina T. N. Abukova L. A. Tourova T. P. Babich T. L. Bidzhieva S. K. Loiko N. G.. (2023). Biodiversity and potential activity of microorganisms in underground gas storage horizons. Sustainability 15, 1–20. doi: 10.3390/su15139945

[ref60] Ollivier B. Fardeau M.-L. Cayol J.-L. Magot M. Patel B. K. C. Prensier G.. (1998). *Methanocalculus halotolerans* gen. Nov., sp. Nov., isolated from an oil-producing well. Int. J. Syst. Bacteriol. 48, 821–828. doi: 10.1099/00207713-48-3-821 9734036

[ref61] Osburn M. R. LaRowe D. E. Momper L. M. Amend J. P. (2014). Chemolithotrophy in the continental deep subsurface: Sanford underground research facility (SURF), USA. Front. Microbiol. 5:610. doi: 10.3389/fmicb.2014.00610 25429287 PMC4228859

[ref62] Perera M. S. A. (2023). A review of underground hydrogen storage in depleted gas reservoirs: insights into various rock-fluid interaction mechanisms and their impact on the process integrity. Fuel 334:126677. doi: 10.1016/j.fuel.2022.126677

[ref63] Philips J. Monballyu E. Georg S. De Paepe K. Prévoteau A. Rabaey K.. (2019). An Acetobacterium strain isolated with metallic iron as electron donor enhances iron corrosion by a similar mechanism as *Sporomusa sphaeroides*. FEMS Microbiol. Ecol. 95, 1–13. doi: 10.1093/femsec/fiy222 30445447

[ref64] Pichler M. Coskun Ö. K. Ortega-Arbulú A. Conci N. Wörheide G. Vargas S.. (2018). A 16S rRNA gene sequencing and analysis protocol for the Illumina MiniSeq platform. MicrobiologyOpen 7:e00611. doi: 10.1002/mbo3.611 29575567 PMC6291791

[ref65] Postgate J. R. (1984). The sulphate-reducing bacteria 2nd ed. Cambridge: Cambridge University Press.

[ref66] Procópio L. (2022). Microbially induced corrosion impacts on the oil industry. Arch. Microbiol. 204:138. doi: 10.1007/s00203-022-02755-7 35032195

[ref67] Purkamo L. Kietäväinen R. Miettinen H. Sohlberg E. Kukkonen I. Itävaara M.. (2018). Diversity and functionality of archaeal, bacterial and fungal communities in deep Archaean bedrock groundwater. FEMS Microbiol. Ecol. 94:fiy116. doi: 10.1093/femsec/fiy116 29893836

[ref68] Ranchou-Peyruse M. Guignard M. Casteran F. Abadie M. Defois C. Peyret P.. (2021). Microbial diversity under the influence of natural gas storage in a deep aquifer. Front. Microbiol. 12:688929. doi: 10.3389/fmicb.2021.688929 34721313 PMC8549729

[ref69] Rempfert K. R. Miller H. M. Bompard N. Nothaft D. Matter J. M. Kelemen P.. (2017). Geological and geochemical controls on subsurface microbial life in the Samail Ophiolite, Oman. Front. Microbiol. 8:56. doi: 10.3389/fmicb.2017.00056 28223966 PMC5293757

[ref70] Sansupa C. Wahdan S. F. M. Hossen S. Disayathanoowat T. Wubet T. Purahong W. (2021). Can we use functional annotation of prokaryotic taxa (FAPROTAX) to assign the ecological functions of soil bacteria? Appl. Sci. 11:688. doi: 10.3390/app11020688

[ref71] Schwab L. Popp D. Nowack G. Bombach P. Vogt C. Richnow H. H. (2022). Structural analysis of microbiomes from salt caverns used for underground gas storage. Int. J. Hydrog. Energy 47, 20684–20694. doi: 10.1016/j.ijhydene.2022.04.170

[ref72] Shlimon A. G. (2004). *Methanobacterium aarhusense* sp. Nov., a novel methanogen isolated from a marine sediment (Aarhus Bay, Denmark). Int. J. Syst. Evol. Microbiol. 54, 759–763. doi: 10.1099/ijs.0.02994-0 15143021

[ref73] Šmigáň P. Greksák M. Kozánková J. Buzek F. Onderka V. Wolf I. (1990). Methanogenic bacteria as a key factor involved in changes of town gas stored in an underground reservoir. FEMS Microbiol. Lett. 73, 221–224. doi: 10.1111/j.1574-6968.1990.tb03944.x

[ref74] Sokolova D. S. Semenova E. M. Grouzdev D. S. Bidzhieva S. K. Babich T. L. Loiko N. G.. (2021). Sulfidogenic microbial communities of the Uzen high-temperature oil field in Kazakhstan. Microorganisms 9:1818. doi: 10.3390/microorganisms9091818 34576714 PMC8467725

[ref75] Sorokin D. Y. Merkel A. Y. (2022). “Dethiobacteria class. Nov” in Bergey’s manual of systematics of Archaea and bacteria (1. Ed., p. 1–3). eds. Trujillo M. E. Dedysh S. DeVos P. Hedlund B. Kämpfer P. Rainey F. A.. (Hoboken, New Jersey: Wiley).

[ref76] Stetter K. O. Thomm M. Winter J. Wildgruber G. Huber H. Zillig W.. (1981). *Methanothermus fervidus*, sp. Nov., a novel extremely thermophilic methanogen isolated from an Icelandic hot spring. Zentralblatt für Bakteriologie Mikrobiologie und Hygiene 2, 166–178. doi: 10.1016/S0721-9571(81)80038-5

[ref77] Strobel G. Hagemann B. Lüddeke C. T. Ganzer L. (2023). Coupled model for microbial growth and phase mass transfer in pressurized batch reactors in the context of underground hydrogen storage. Front. Microbiol. 14:1150102. doi: 10.3389/fmicb.2023.1150102 37082185 PMC10110988

[ref78] Suzuki D. Li Z. Cui X. Zhang C. Katayama A. (2014). Reclassification of *Desulfobacterium anilini* as Desulfatiglans anilini comb. Nov. within Desulfatiglans gen. Nov., and description of a 4-chlorophenol-degrading sulfate-reducing bacterium, Desulfatiglans parachlorophenolica sp. Nov. Int. J. Syst. Evol. Microbiol. 64, 3081–3086. doi: 10.1099/ijs.0.064360-0 24944334

[ref79] Thaysen E. M. McMahon S. Strobel G. J. Butler I. B. Ngwenya B. T. Heinemann N.. (2021). Estimating microbial growth and hydrogen consumption in hydrogen storage in porous media. Renew. Sust. Energ. Rev. 151:111481. doi: 10.1016/j.rser.2021.111481

[ref80] Tremosa J. Jakobsen R. Le Gallo Y. (2023). Assessing and modeling hydrogen reactivity in underground hydrogen storage: a review and models simulating the Lobodice town gas storage. Front. Energy Res. 11:1145978. doi: 10.3389/fenrg.2023.1145978

[ref81] Uchiyama T. Ito K. Mori K. Tsurumaru H. Harayama S. (2010). Iron-corroding methanogen isolated from a crude-oil storage tank. Appl. Environ. Microbiol. 76, 1783–1788. doi: 10.1128/AEM.00668-09 20118376 PMC2838011

[ref82] Vigneron A. Alsop E. B. Lomans B. P. Kyrpides N. C. Head I. M. Tsesmetzis N. (2017b). Succession in the petroleum reservoir microbiome through an oil field production lifecycle. ISME J. 11, 2141–2154. doi: 10.1038/ismej.2017.78 28524866 PMC5563965

[ref83] Vigneron A. Bishop A. Alsop E. B. Hull K. Rhodes I. Hendricks R.. (2017a). Microbial and isotopic evidence for methane cycling in hydrocarbon-containing groundwater from the Pennsylvania region. Front. Microbiol. 8:593. doi: 10.3389/fmicb.2017.00593 28424678 PMC5380731

[ref84] Vítězová M. Onderka V. Urbanová I. Molíková A. Hanišáková N. Buriánková I.. (2023). In situ field experiment shows the potential of methanogenic archaea for biomethane production from underground gas storage in natural rock environment. Environ. Technol. Innovat. 32:103253. doi: 10.1016/j.eti.2023.103253

[ref85] Watanabe S. Hamano M. Kakeshita H. Bunai K. Tojo S. Yamaguchi H.. (2003). Mannitol-1-phosphate dehydrogenase (MtlD) is required for Mannitol and Glucitol assimilation in *Bacillus subtilis*: possible cooperation of mtl and gut operons. J. Bacteriol. 185, 4816–4824. doi: 10.1128/JB.185.16.4816-4824.2003, PMID: 12897001 PMC166460

[ref86] Weng C.-Y. Chen S.-C. Lai M.-C. Wu S.-Y. Lin S. Yang T. F.. (2015). *Methanoculleus taiwanensis* sp. Nov., a methanogen isolated from deep marine sediment at the deformation front area near Taiwan. Int. J. Syst. Evol. Microbiol. 65, 1044–1049. doi: 10.1099/ijs.0.000062 25575827

[ref87] Widdel F. Bak F. (1992). “Gram-negative Mesophilic sulfate-reducing bacteria” in The prokaryotes. eds. Balows A. Trüper H. G. Dworkin M. Harder W. Schleifer K.-H. (New York: Springer), s. 3352–3378.

[ref88] Worakit S. Boone D. R. Mah R. A. Abdel-Samie M.-E. El-Halwagi M. M. (1986). *Methanobacterium alcaliphilum* sp. Nov., an H_2_-utilizing methanogen that grows at high pH values. Int. J. Syst. Bacteriol. 36, 380–382. doi: 10.1099/00207713-36-3-380

[ref89] Wulf C. Zapp P. Schreiber A. (2020). Review of power-to-X demonstration projects in Europe. Front. Energy Res. 8:191. doi: 10.3389/fenrg.2020.00191

[ref90] Xu X. Liu W. Tian S. Wang W. Qi Q. Jiang P.. (2018). Petroleum hydrocarbon-degrading bacteria for the remediation of oil pollution under aerobic conditions: a perspective analysis. Front. Microbiol. 9:2885. doi: 10.3389/fmicb.2018.02885 30559725 PMC6287552

[ref91] Yang C. Mai J. Cao X. Burberry A. Cominelli F. Zhang L. (2023). ggpicrust2: An R package for PICRUSt2 predicted functional profile analysis and visualization. arXiv [Preprint]. doi: 10.48550/arXiv.2303.10388 PMC10425198 37527009

[ref92] Yang Z. Peng C. Cao H. Song J. Gong B. Li L.. (2022). Microbial functional assemblages predicted by the FAPROTAX analysis are impacted by physicochemical properties, but C, N and S cycling genes are not in mangrove soil in the Beibu Gulf, China. Ecol. Indicat. 139:108887. doi: 10.1016/j.ecolind.2022.108887

[ref93] Zhang Y. Dekas A. E. Hawkins A. J. Parada A. E. Gorbatenko O. Li K.. (2019). Microbial community composition in deep-subsurface reservoir fluids reveals natural interwell connectivity. Water Resour. Res. 56:e2019WR025916. doi: 10.1029/2019WR025916

[ref94] Zhilina T. N. Zavarzina D. G. Kevbrin V. V. Kolganov T. V. (2013). *Methanocalculus natronophilus* sp. Nov., a new alkaliphilic hydrogenotrophic methanogenic archaeon from a soda lake, and proposal of the new family Methanocalculaceae. Mikrobiologiia 82, 681–690. doi: 10.1134/S0026261713060131 25509406

