# Supplementary material for: Microbiological insight into various underground gas storages in Vienna Basin focusing on methanogenic Archaea
Source: Front Microbiol. 2023 Dec 13;14:1293506. doi: 10.3389/fmicb.2023.1293506 (PMC10771303; doi:10.3389/fmicb.2023.1293506)
Supplement: Supplementary file 1 [file Data_Sheet_1.pdf]

Supplementary Material

Microbiological insight into various underground gas storages in Vienna Basin focusing on methanogenic *Archaea*

Nikola Hanišáková, Monika Vítězová, Tomáš Vítěz*, Ivan Kushkevych, Eva Kotrlová, David Novák, Jan Lochman, Roman Zavada

*** Correspondence:** Tomáš Vítěz: vitez@sci.muni.cz

Table S1 Medium for SRB

	**Postgate C** (g/L)
KH_2PO_4	0.5
NH_4Cl	1
Na_2SO_4	4.5
$CaCl_2.6H_2O$	0.06
$MgSO_4.7H_2O$	0.06
$FeSO_4.7H_2O$	0.004
sodium citrate.$2H_2O$	0.3
sodium lactate	6
yeast extract	1
ascorbic acid	0.1
Mohr salt	1

The compounds, emitting ascorbic acid and Mohr salts, were dissolved in deionised water and filled to 1L. The Mohr salt was dissolved in 10 ml of deionized water and autoclaved, as well as medium (121°C, 15 min). In aseptic conditions, ascorbic acid was dissolved in deionized water and added through filter 0.22µm Isopore^{tm} (Merck, Darmstadt, Germany) into medium. Mohr salt solution was also transferred to the medium and pH of the medium was adjusted to 7. The medium is ready to use.

Table S2 Cultivation media for methanogens

	MM (g/L)	SW (g/L)	DSMZ 141 (g/L)
KCl	0.5	0.5	0.34
$MgCl_2.6H_2O$	0.4	3	4
$MgSO_4.7H_2O$	-	-	3.45
NH_4Cl	0.25	0.25	0.28
$CaCl_2.2H_2O$	0.1	0.15	0.14
KH_2PO_4	0.2	0.2	-
K_2HPO_4	-	-	0.14
NaCl	1	20	18
$NaHCO_3$	2.5	2.5	4
Yeast extract	-	-	2
Tryptone	-	-	2
$Fe(NH_4)_2(SO_4)_2$ x $6H_2O$ sol. (0.1% w/v)	-	-	2 ml
Sodium acetate	0.01	0.01	1
Methanol 99%	0.02	0.02	-
Na_2S (0.5M)	6 ml	6 ml	6 ml
L-cysteine	0.001	0.001	0.001
Trace elements SL10[a]	1 ml	1 ml	-
Selenite-tungstate[b]	1 ml	1 ml	1.2 ml
Vitamin solution[c]	1 ml	1 ml	-
Wolfe's mineral solution[d]	-	-	1 ml
Wolfe's vitamin solution[e]	-	-	10 ml

[a]concentration in medium mg/L: $FeCl_2 \cdot 6H_2O$ 1; $ZnCl_2$ 0.07; $MnCl_2 \cdot 2H_2O$ 0.1; H_3BO_4 0.006; $CoCl_2 \cdot 6H_2O$ 0.13; $CuCl_2 \cdot 2H_2O$ 0.002; $NiCl_2 \cdot 6H_2O$ 0.024; g $Na_2MoO_4 \cdot 2H_2O$ 0.036

[b]concentration in medium µg/L: $Na_2WO_4 \cdot 2H_2O$ 3.3 µg; $Na_2SeO_3 \cdot 5H_2O$ 2.63 µg

[c]concentration in medium µg/L: riboflavin 50; thiamine-hydrochloride 100; biotin 10; pyridoxin-dihydrochloride 150; nicotinic a. 100; calcium-D(+)-pantothenate 50; 4-aminobenzoic acid 40; cyanocobalamin 50

[d]concentration in medium mg/L: $MgSO_4 \cdot 7H_2O$ 30; $MnSO_4 \cdot H_2O$ 5; NaCl 10; $FeSO_4 \cdot 7H_2O$ 1; $CoCl_2 \cdot 6H_2O$ 1.8; $CaCl_2 \cdot 2H_2O$ 1; $ZnSO_4 \cdot 7H_2O$ 1.8; $CuSO_4 \cdot 5H_2O$ 0.1; $KAl(SO_4)_2 \cdot 12H_2O$ 0.18; H_3BO_4 0.1; $Na_2MoO_4 \cdot 2H_2O$ 1.2; $(NH_4)_2Ni(SO_4)_2 \cdot 6H_2O$ 2.8; $Na_2WO_4 \cdot 2H_2O$ 1.2; $Na_2SeO_3.5H_2O$ 0.14

[e]concentration in medium µg/L: pyridoxin-hydrochloride 10; thiamine-hydrochloride 5; riboflavin 5; nicotinic a. 5; calcium-D-(+)-pantothenate 5; 4-paraaminobenzoic a. 5; lipoic a. 5; biotin 2; folic a. 2; cyanocobalamin 0.1

The compounds were dissolved in deionized water, expect for vitamin solutions, L-cysteine, Na_2S and methanol, filled to 1L and pH adjusted to 7. 30 ml of the medium was dispersed into serum bottles, that were closed with butyl stopper and secured with aluminium cap. The gas phase in serum bottles was exchanged to H_2:CO_2 (4:1 v/v) by repeating cycle vacuum-gas seven times (MZ 2C NT, Vacuubrand, Germany) and autoclaved (121°C, 15 min). The vitamin solution, L-cysteine Na_2S and methanol were injected to serum bottles to reach respective concentrations.

Table S3 Alpha diversity of the studied samples from deep and surface environment.

	Observed	Chao1	se.chao1	ACE	se.ACE	Shannon	Simpson	InvSimpson	Fisher	Coverage
S1	271	287.65	7.363	294.399	8.441	1.766	0.658	2.921	35.381	0.999507
S2	296	443.86	69.043	375.543	8.386	3.384	0.880	8.329	39.164	0.999387
S4	176	214.75	18.805	205.287	7.154	1.893	0.595	2.470	21.586	0.999587
S5	340	370.49	11.577	372.568	9.560	2.348	0.669	3.021	45.958	0.99936
S6	386	456.04	23.804	428.823	10.205	3.392	0.924	13.148	53.232	0.999173
R7	96	120	16.419	108.022	4.952	1.707	0.702	3.357	10.859	0.999787
D1	340	413.2	25.222	390.732	9.688	2.524	0.751	4.021	45.958	0.999187
D2	215	236	10.463	232.705	7.556	2.881	0.903	10.295	27.129	0.999627
D3	169	192	12.763	183.502	6.559	2.825	0.890	9.066	20.610	0.999693
D4	291	346	19.472	339.950	9.172	3.023	0.884	8.599	38.403	0.999253
D5	570	665	25.673	653.105	12.597	3.881	0.934	15.233	83.858	0.99872
D6	277	338.5	26.102	304.627	8.620	2.444	0.772	4.394	36.283	0.99944
D7	247	284.05	15.711	279.597	8.367	3.200	0.903	10.284	31.805	0.99948

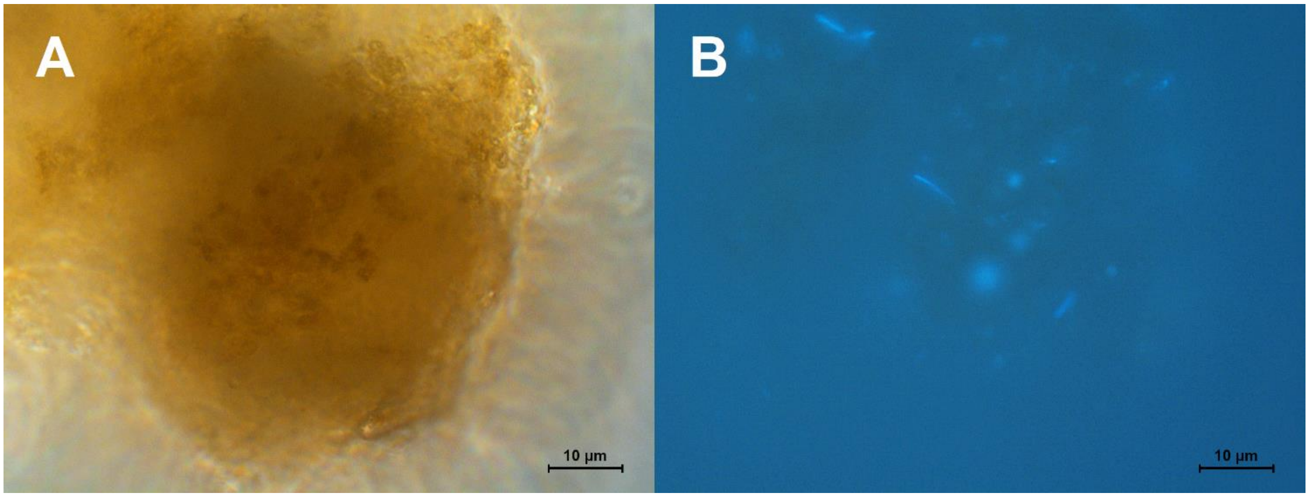

Figure S1. Native preparation of the corrosive precipitate of S1 sample in bright field **(A)** and under the UV filter **(B)**. Autofluorescent rods and cocci are visible as part of the precipitate.

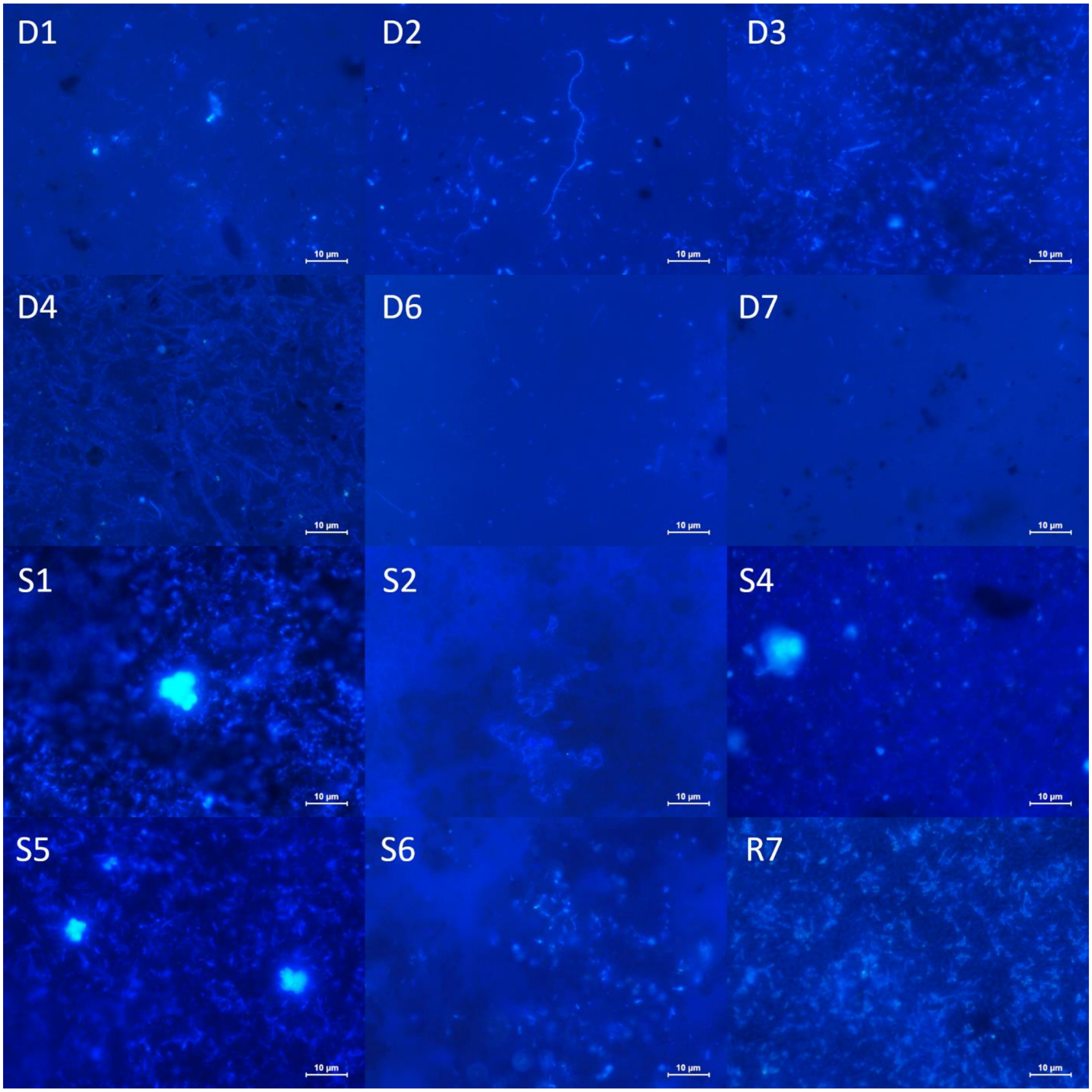

Figure S2. Images of microbial cells in individual samples, stained with the fluorescent dye DAPI. Magnification 1000x. The light bundles in the sample S1, S4 and S5 are cells of *Methanosarcina* species.

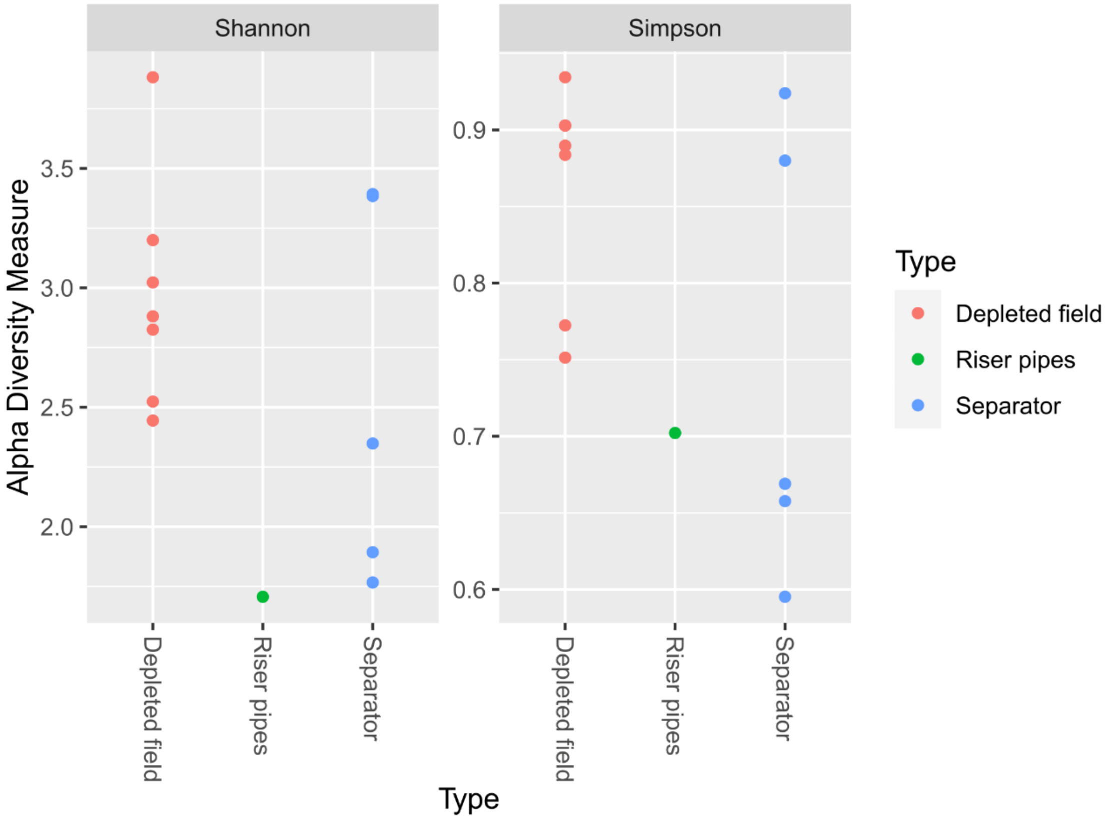

Figure S3. Alfa-diversity of samples calculated using Shannon and Simpson index, differentiated according to type of the sample.

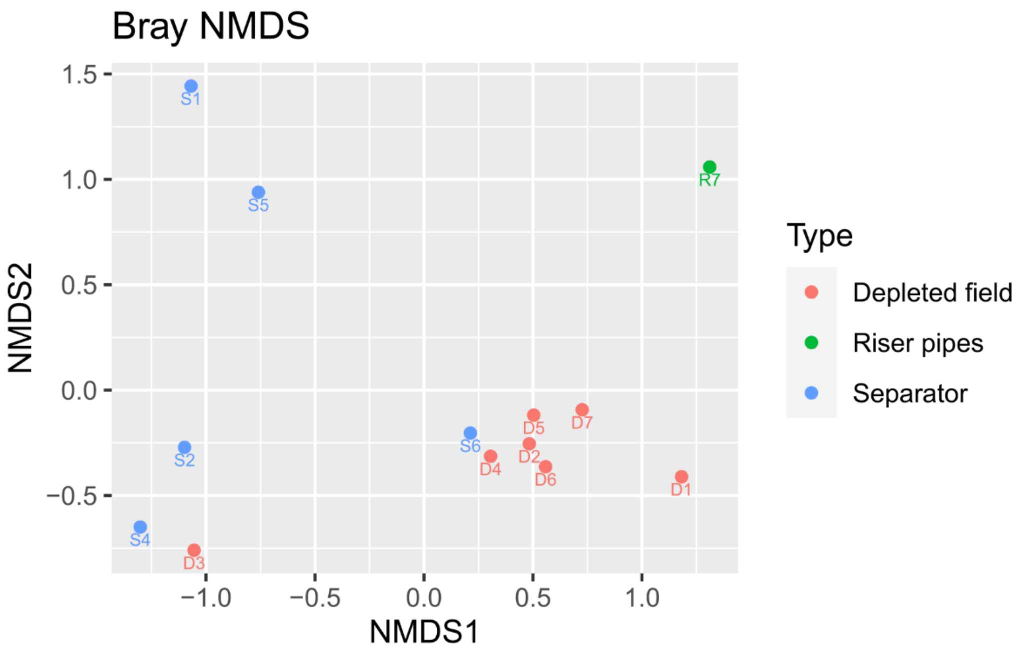

Figure S4. Beta-diversity of samples calculated using NMDS Bray-Curtis distance method

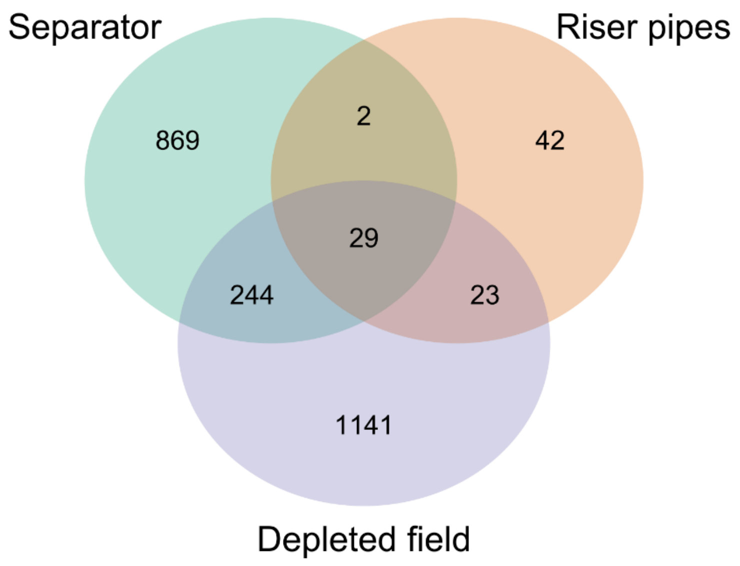

Figure S5. Venn diagram of showcasing unique taxa between the type of samples, divided to Separator, Riser pipes and Depleted field.

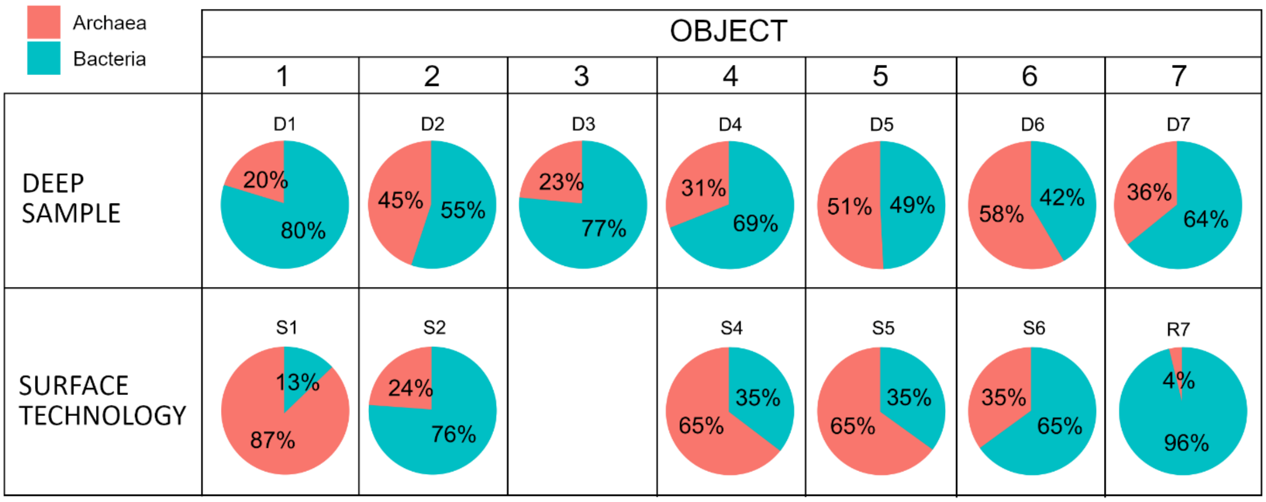

Figure S6. Percentual ratio of archaeal and bacterial 16S rRNA in each sample. Samples are subdivided by the object from which they were collected (column) and by the type of sample (row).

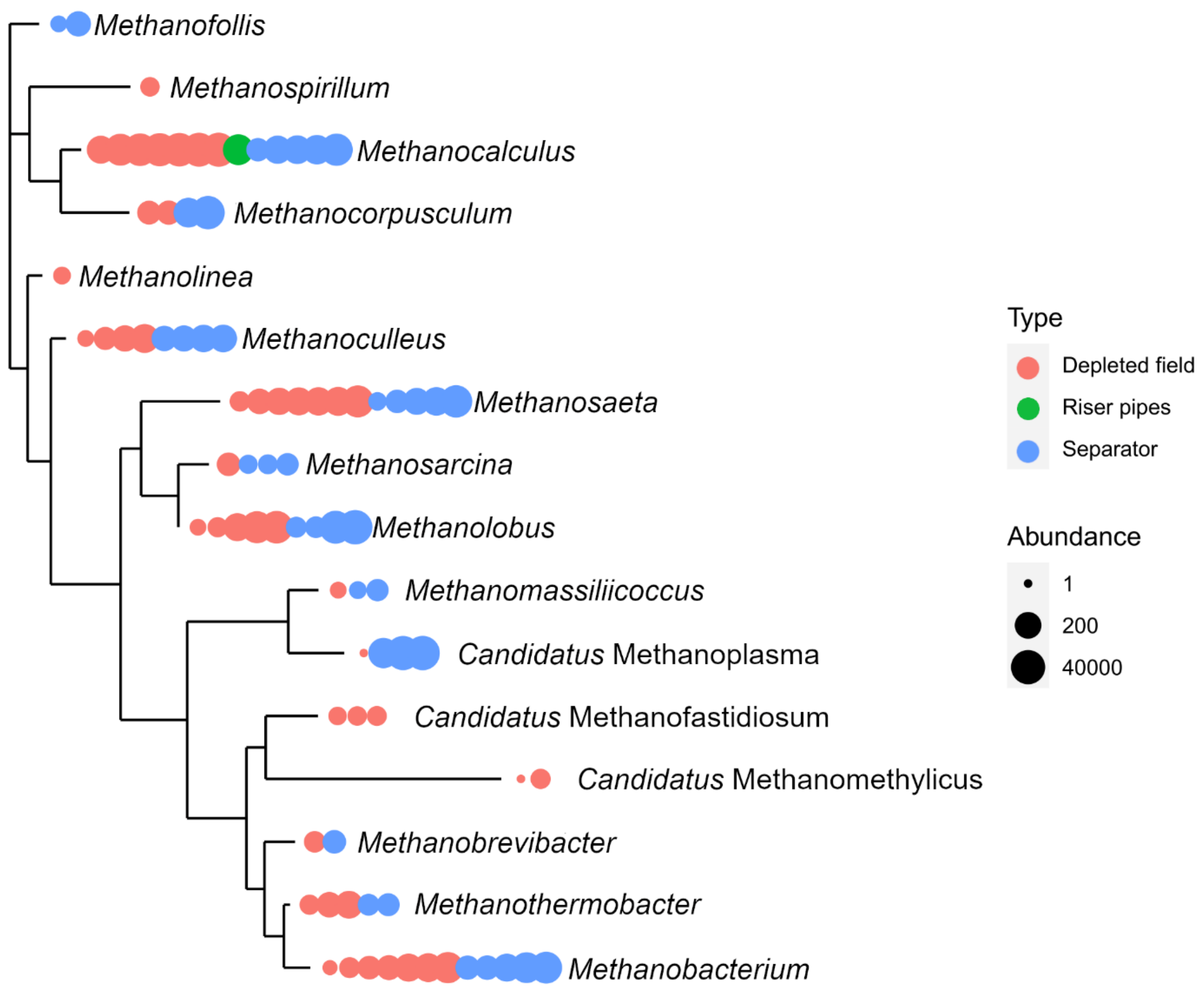

Figure S7. Phylogenetic tree of methanogens on genus level constructed from sequenced data of samples. The dots showcase the abundance of present genera in various types of samples.

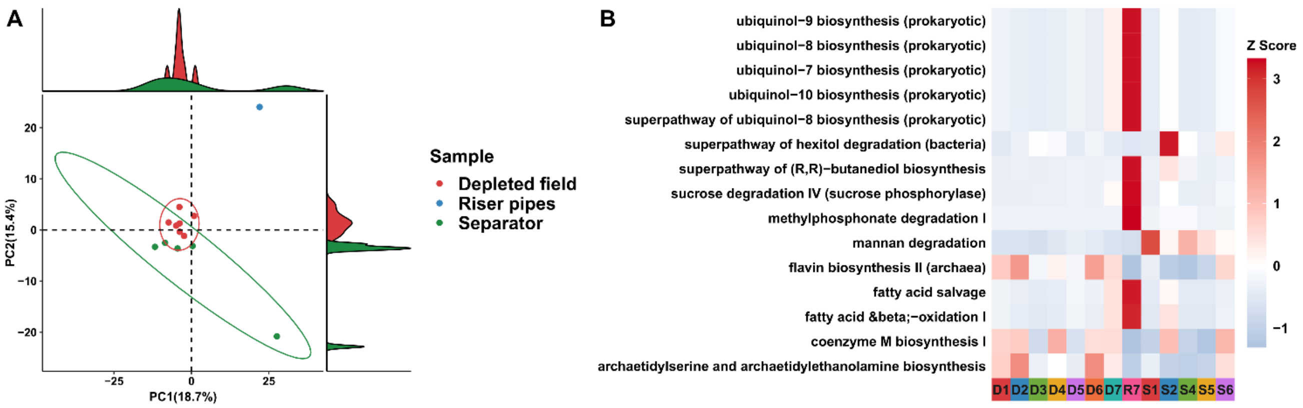

Figure S8. Predicted MetaCyc metabolic pathways PICRUSt2. **(A)** PCoA plot constructed based on the abundance of the metabolic pathways analysed, showing the clustering of the different sample types. **(B)** Differential abundances of MetaCyc pathways for all three groups – deep sample, separator and riser pipes, calculated by ALDEx2 method, p value adjusted by FDR, showing only statistically significant pathways ($p<0.05$).

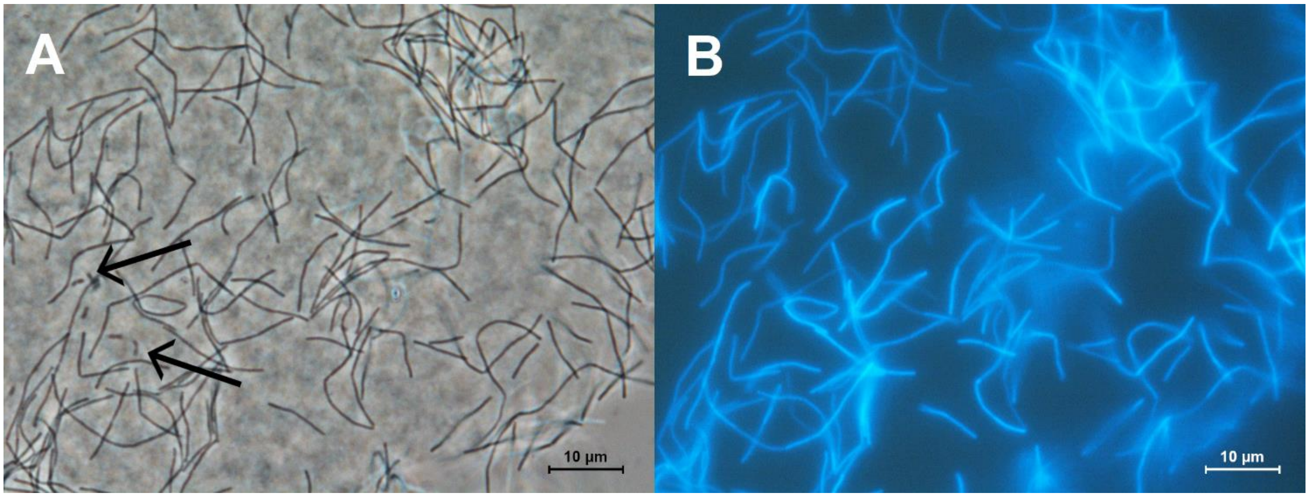

Figure S9. The native sample preparation of *Methanothermobacter* sp. and *Pseudothermotoga* sp. from sample D7 (1000x) observed in bright field **(A)** and under UV filter **(B)**. The arrows show the cells of *Pseudothermotoga* sp. in the matrix created probably by methanogens.

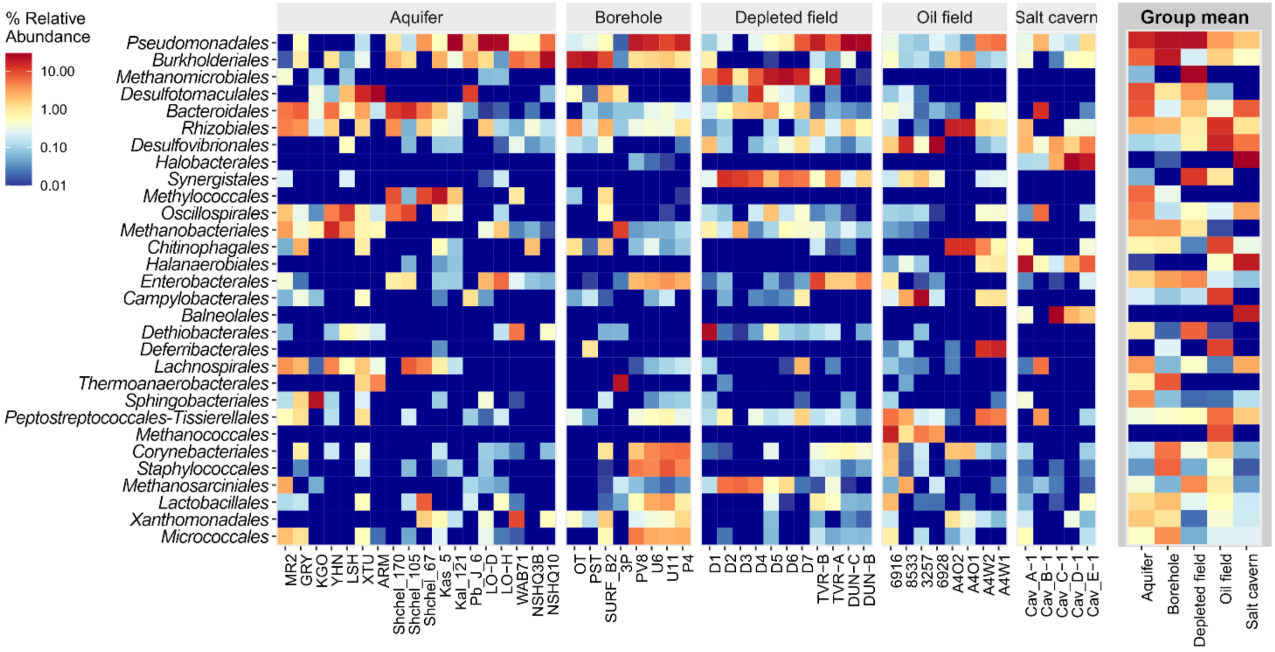

Figure S10. Metanalysis of terrestrial subsurface samples of **Set 1**: The relative abundance of the thirty most abundant microbial classes from all samples, separated according the underground sample type, with special group mean column showing mean abundance of the orders, separated by a type of sample.

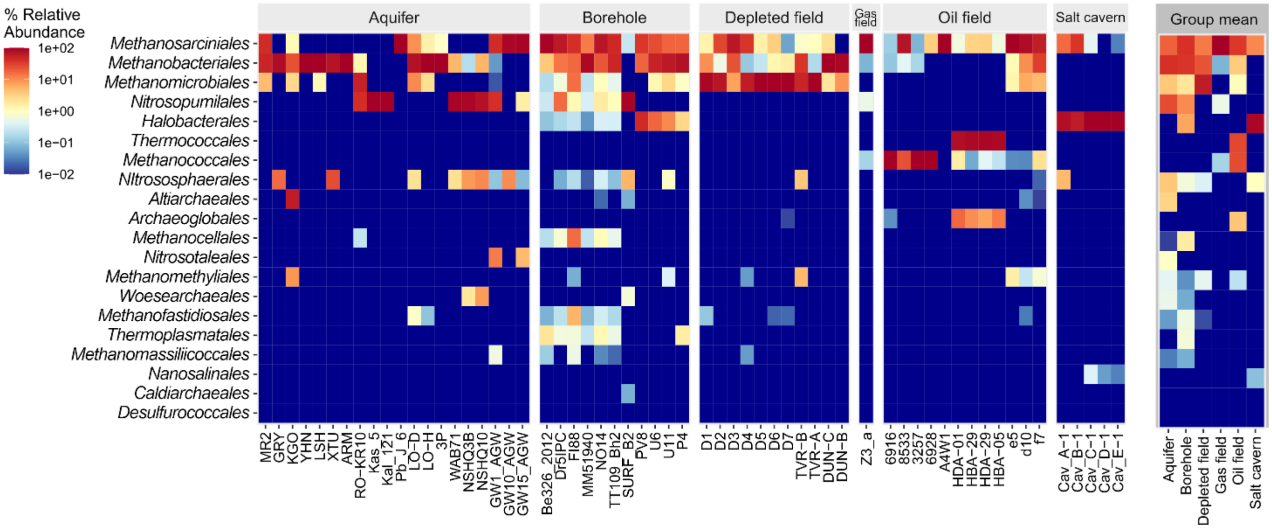

Figure S11. The relative abundance of genera from all samples of **Set 3**, containing only species of the domain *Archaea*. Separated according the underground sample type.

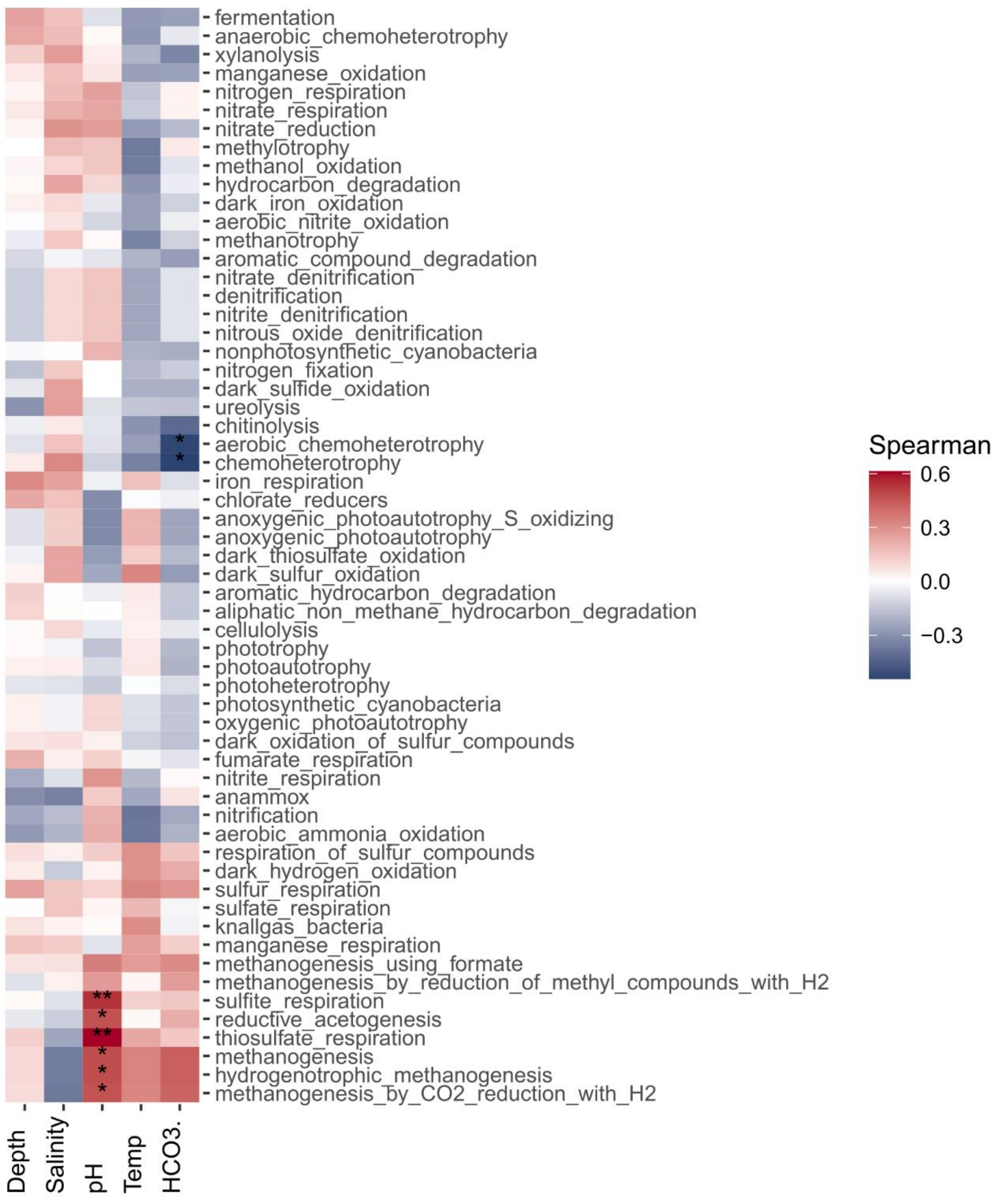

Figure S12. Metanalysis of terrestrial subsurface samples in **Set 1**: Spearman's correlation of functional metabolic prediction FAPROTAX and physical parameters of the samples (* $p < 0.05$, ** $p < 0.01$, *** $p < 0.001$)

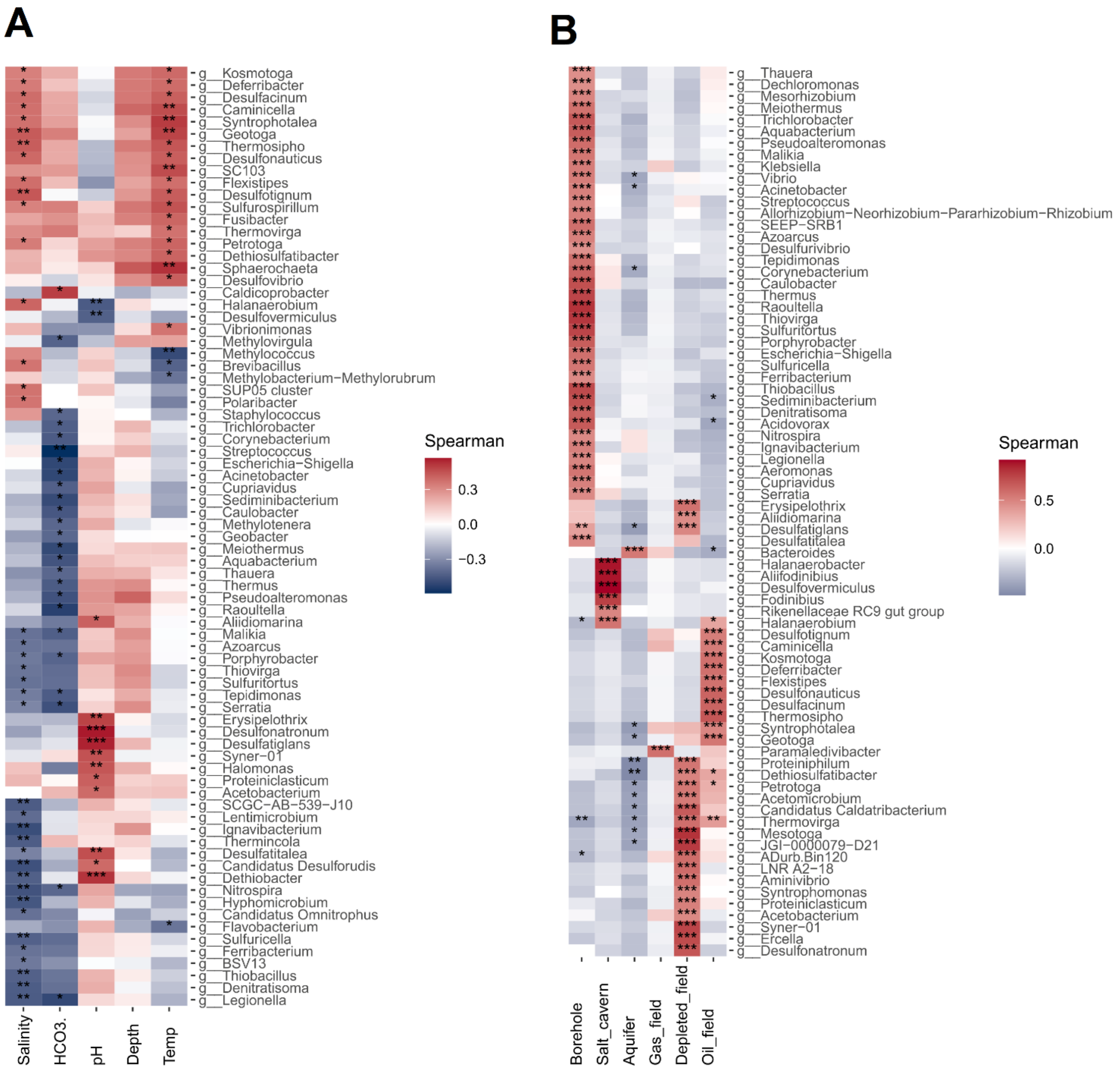

Figure S13. Metanalysis of terrestrial subsurface samples in **Set 2**: Spearman's corelation heatmap of microbial genera and different underground conditions **(A)** and types of sample **(B)**, showed only corelation of first 200 most abundant taxa, and statistically significant taxa (* $p < 0.05$, ** $p < 0.01$, *** $p < 0.001$). P value was adjusted by FDR method.

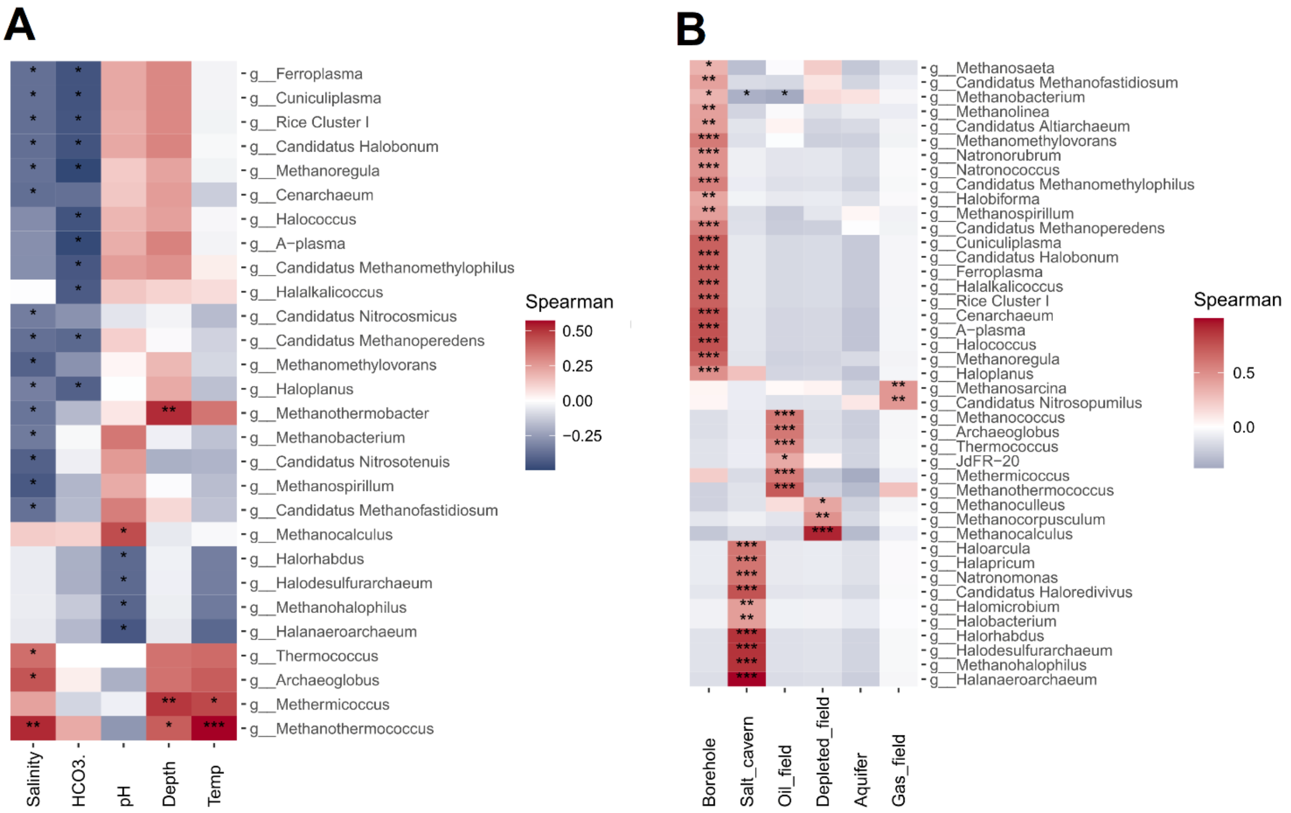

Figure S14. Metanalysis of terrestrial subsurface samples in **Set 3**: Spearman's correlation matrix of archaeal genera and environmental parameters **(A)** or different sample types **(B)** showed only statistically significant taxa (* $p < 0.05$, ** $p < 0.01$, *** $p < 0.001$). P value was adjusted by FDR method.
